# On a Factorization Formula for the Partition Function of Directed Polymers

**DOI:** 10.1007/s10955-023-03172-w

**Published:** 2023-10-20

**Authors:** Tobias Hurth, Konstantin Khanin, Beatriz Navarro Lameda, Fedor Nazarov

**Affiliations:** 1 Institute for Mathematics, Freie Universität Berlin, Arnimallee 9, 14195 Berlin, Germany; 2 Department of Mathematics, University of Toronto, Bahen Centre, 40 St. George Street, Toronto, ON M5S 2E4 Canada; 3 Department of Mathematics, University College London, Gower Street, London, WC1E 6BT UK; 4 Department of Mathematical Sciences, Kent State University, E. Summit Street, Kent, OH 44242 USA

**Keywords:** Directed polymers, Random walk in a random environment, Weak disorder, Partition function

## Abstract

We prove a factorization formula for the point-to-point partition function associated with a model of directed polymers on the space-time lattice $\mathbb {Z}^{d+1}$. The polymers are subject to a random potential induced by independent identically distributed random variables and we consider the regime of weak disorder, where polymers behave diffusively. We show that when writing the quotient of the point-to-point partition function and the transition probability for the underlying random walk as the product of two point-to-line partition functions plus an error term, then, for large time intervals [0, *t*], the error term is small uniformly over starting points *x* and endpoints *y* in the sub-ballistic regime $\Vert x - y \Vert \le t^{\sigma }$, where $\sigma < 1$ can be arbitrarily close to 1. This extends a result of Sinai, who proved smallness of the error term in the diffusive regime $\Vert x - y \Vert \le t^{1/2}$. We also derive asymptotics for spatial and temporal correlations of the field of limiting partition functions.

## Introduction

The theory of directed polymers has been actively studied in the mathematical and physical literature in the last 30 years. From the point of view of probability theory and statistical mechanics, directed polymers are random walks in a random potential. The probability distribution for a random path $\gamma$ of length *t* is given by the Gibbs distribution $P^t_\omega (\gamma )= \frac{1}{Z^t_\omega }\exp {\left[ -\beta H^t_\omega (\gamma )\right] }$, where $\beta$ is the inverse temperature, $H^t_\omega (\gamma )$ is the total energy of the interaction between the path $\gamma$ and a fixed realization of the external random potential, and the normalizing factor $Z^t_\omega$ is the partition function. The random potential is a functional defined on some probability space, and a point $\omega$ in this probability space completely characterizes a fixed realization of the potential. In this paper we are interested only in the case of non-stationary time-dependent random potentials. The simplest setting corresponds to the discrete space-time lattice $\mathbb {Z}^{d+1}$, where *d* is the spatial dimension. In this case the random potential normally is assumed to be given by the i.i.d. field $\omega =\{\xi (x,i): \, x\in \mathbb {Z}^d, i\in \mathbb {Z}\}$, and $H_\omega ^t= -\sum _{i=0}^t {\xi (\gamma _i,i)}$. As usual one is interested in the asymptotic behavior of directed polymers as $t\rightarrow \infty$.

The first rigorous results for directed polymers were obtained by Imbrie and Spencer [1], Bolthausen [2], and Sinai [3]. It was proved that in the case of weak disorder, namely when $d \ge 3$ and $|\beta |$ is small, the polymer almost surely has diffusive behavior with a non-random covariance matrix. It was later proved by Carmona and Hu [4], and Comets et al. [5] that in the cases $d=1,2$, and $d\ge 3$ with $|\beta |$ large, the asymptotic behavior is very different. In this regime, called strong disorder, the directed polymers are not spreading as $t\rightarrow \infty$ but remain concentrated in certain random places.

Sinai’s approach in [3] is based on the study of asymptotic properties of partition functions $Z^t_\omega$ as $t \rightarrow \infty$. It turns out that if the polymer starts at a point *x* at time *s*, then in the limit $t\rightarrow \infty$ the properly normalized partition function converges almost surely to a random variable $Z^\infty _{x,s}$. Here, in order to simplify notation, we are not indicating the dependence on $\omega$. In a similar way one can consider backward in time partition functions, and prove that after the same normalization they also converge to limiting partition functions $Z_{-\infty }^{y,t}$, where (*y*, *t*) is the endpoint of the polymer. The proof of the diffusive behavior follows from a factorization formula proved by Sinai. Namely, a bridging partition function $Z_{x,s}^{y,t}$ corresponding to the random-walk bridge between points $(x,s), \, (y,t), \, t>s,$ satisfies the following asymptotic relation:

1 $$\begin{aligned} Z_{x,s}^{y,t}= q^{y-x}_{t-s}(Z_{x,s}^{\infty } Z_{-\infty }^{y,t} +\delta _{x,s}^{y,t}), \end{aligned}$$

where $q^{y-x}_{t-s}$ is the transition probability of the simple symmetric random walk, and a small error term $\delta _{x,s}^{y,t}$ tends to zero as $t-s \rightarrow \infty$, provided $y-x$ belongs to the diffusive region: $\Vert y-x\Vert =O(\sqrt{t-s})$. Later, Sinai’s formula was extended by Kifer [6] to the continuous setting.

The interest in the asymptotic behavior of directed polymers is largely motivated by the connection between directed polymers and the theory of the stochastic heat equation

$$\begin{aligned} \partial _tZ(x,t)=\frac{1}{2} \Delta Z(x,t) + \xi ^\omega (x,t)Z(x,t) \end{aligned}$$

and the random Hamilton-Jacobi equation

$$\begin{aligned} \partial _t \Phi (x,t) + \frac{1}{2} |\nabla \Phi (x,t) |^2 = \frac{1}{2}\Delta \Phi (x,t) - \xi ^\omega (x,t), \end{aligned}$$

which is related to the stochastic heat equation through the Hopf-Cole transformation $\Phi (x,t) = - \ln Z(x,t)$. The connection between directed polymers and the stochastic heat equation is a direct consequence of the Feynman-Kac formula, see, e.g., [7].

The main conjecture about the asymptotic behavior of the solutions to the random Hamilton-Jacobi equation can be formulated in the following way. For a fixed value of the average velocity $b=\langle \nabla \Phi (x, \cdot )\rangle$, which is preserved by the equation, with probability one there exists a unique (up to an additive constant) global solution. This means that solutions starting from two different initial conditions $\Phi _1(x,0)=b\cdot x +\Psi _1(x,0), \, \Phi _2(x,0)=b\cdot x +\Psi _2(x,0)$ approach each other up to an additive constant as $t\rightarrow \infty$, provided $\Psi _1(x,0)$ and $\Psi _2(x,0)$ are functions of sublinear growth in $\Vert x\Vert$ [7].

In terms of the stochastic heat equation, a similar uniqueness statement up to a multiplicative constant conjecturally holds for two initial conditions of the form $Z_1(x,0)=\exp {[-b\cdot x -\Psi _1(x,0)]}$ and $Z_2(x,0)=\exp {[-b\cdot x -\Psi _2(x,0)]}$.

In order to be able to prove the above conjecture in the weak-disorder case, one has to extend the factorization formula (1) to a much larger scale. This is the purpose of the present article: We prove that the factorization formula holds for $\Vert x-y\Vert <(t-s)^\sigma$, where $\sigma$ can be taken arbitrarily close to 1. Compared to [3], such an extension of the factorization formula requires very different analytical methods.

In this paper we restrict ourselves to the simplest discrete case, i.e., polymers live on the discrete space-time lattice $\mathbb {Z}^{d+1}$ and the potential is induced by an i.i.d. field of random variables. This allows us to make the exposition more transparent. However, in order to prove the uniqueness conjecture for the stochastic heat equation in the weak-disorder regime, one needs to consider the parabolic Anderson model, which is discrete in space and continuous in time. The proof of the factorization formula in this semi-discrete setting, which is based on similar ideas but technically more involved, will be published elsewhere. The proof of the uniqueness conjecture itself will be published separately as well.

We conclude the introduction with several remarks:

1. The factorization formula can be extended to the full sub-ballistic regime $\Vert x-y\Vert =o(t-s)$. We are considering a smaller region $\Vert x-y\Vert <(t-s)^\sigma$, which allows for effective estimates of the smallness of the error term $\delta _{x,s}^{y,t}$.
2. We believe that a similar factorization formula can be proved in the fully continuous case. For this, one would need to assume that the correlations of the disorder field $\{\xi (x,t): x\in \mathbb {R}^d, t \in \mathbb {R}\}$ are decaying sufficiently fast.
3. It is interesting to study the probability distribution for the limiting partition function $Z := Z_{x,s}^{\infty }$ and for $\Phi =-\ln {Z}$. Although these probability distributions are not universal, we believe that the tail distributions have many universal features. We conjecture that in the case when the probability distribution of $\xi$ has compact support, the left tail of the density for $\Phi$ behaves like $\exp {[-\Phi ^{1+d/2}]}$ and the right tail decays like $\exp {[-\Phi ^{1+d}]}$. A related conjecture concerns the moments $m(l) :=\langle Z^l\rangle$ of *Z*, which we conjecture to grow as $\exp {[l^{1+2/d}]}$ in the limit $l\rightarrow \infty$. If the disorder $\xi$ is Gaussian, then for any $\beta >0$ only a finite number of moments for *Z* is finite. Thus, one can expect exponential decay of the left tail for $\Phi$.
4. The uniqueness of global solutions to the stochastic heat equation and the random Hamilton-Jacobi equation was also proved in dimension $d=1$ [8–10]. The mechanism leading to uniqueness in this case is completely different from our setting. We should also mention that the case $d=1$ corresponds to the famous KPZ universality class.

The rest of this paper is organized as follows: In Sect. 2 we derive an expansion for the partition functions and convergence to limiting partition functions. This allows us to state our main result, the factorization formula for $Z_{x,s}^{y,t}$. We also derive asymptotics for both spatial and temporal correlations of the field of limiting partition functions. In Sect. 3, we collect several estimates on transition probabilities for the simple symmetric random walk on $\mathbb {Z}^d$. Sections 4 and 5 are devoted to the proof of the factorization formula. Finally, in the appendix we prove the estimates on transition probabilities from Sect. 3.

**Notation:** Throughout this article the Euclidean norm and inner product in $\mathbb {R}^d$ are denoted by $\Vert \cdot \Vert$ and $$\ \cdot \ $$, respectively. The 1-norm in $\mathbb {R}^d$ is denoted by $\Vert \cdot \Vert _1$. We simply write $a \equiv b$ to indicate that $a \equiv b$ (mod 2). For functions *A* and *B*, potentially of several variables, we write $A \lesssim B$ or *A* is *dominated* by *B* to denote that $A \le c B$ for some constant $c > 0$. The constant *c* may depend on the dimension *d*, the inverse temperature $\beta$ and the law of the disorder (e.g., through $\lambda$ defined in (6)), or on scaling parameters such as $\sigma$ from Theorem 3 or $\xi$ from Sect. 4.1. However, *c* is not allowed to depend on any time or space variables such as *t* and *z*. The same remark applies to every constant introduced in this paper. Finally, in order to simplify notation, we will write $\sum _{\textbf{z}}$ to indicate that we are summing over all $\textbf{z}= (z_1, \ldots , z_r) \in (\mathbb {Z}^d)^r$, where the value of *r* will be clear from the context.

## Setting and Main Result

Let $\gamma = (\gamma _n)_{n \in \mathbb {Z}}$ be a discrete-time simple symmetric random walk on $\mathbb {Z}^d$, $d \ge 3$, starting at point $x \in \mathbb {Z}^d$ at time $s \in \mathbb {Z}$, with corresponding probability measure $\textbf{P}_{x,s}$ and corresponding expectation $\textbf{E}_{x,s}$. As $d \ge 3$, $\gamma$ is transient. For integers $t > s$ and $y \in \mathbb {Z}^d$, we denote the probability measure obtained from $\textbf{P}_{x,s}$ by conditioning on the event $\{\gamma _t = y\}$ by $\textbf{P}_{x,s}^{y,t}$. The corresponding expectation is denoted by $\textbf{E}_{x,s}^{y,t}$. We also set

$$\begin{aligned} q_t^z {:}=\textbf{P}_{0,0}(\gamma _t = z). \end{aligned}$$

Let $(\xi (x, t))_{x \in \mathbb {Z}^d, t \in \mathbb {Z}}$ be a collection of i.i.d. random variables with corresponding probability measure *Q* and corresponding expectation $\langle \,\cdot \, \rangle$. These constitute the random potential in our setting. We assume that

$$\begin{aligned} c(\beta ) := \langle e^{\beta \xi (0,0)} \rangle < \infty \end{aligned}$$

for $\beta > 0$ sufficiently small. To a sample path of $\gamma$ over a time interval [*s*, *t*], we assign the random action

$$\begin{aligned} \mathcal {A}_s^t = \mathcal {A}_s^t (\gamma ) {:}=\sum _{j=s}^t \xi (\gamma _j, j). \end{aligned}$$

For integers $s < t$, $x, y \in \mathbb {Z}^d$, and inverse temperature $\beta > 0$, we define the random partition functions

$$\begin{aligned}{} & {} Z_{x,s}^{y,t} {:}=c(\beta )^{-(t-s+1)} \ q_{t-s}^{y-x} \ \textbf{E}_{x,s}^{y,t} e^{\beta \mathcal {A}_s^t}, \\{} & {} Z_{x,s}^t {:}=\sum _{y \in \mathbb {Z}^d} Z_{x,s}^{y,t}, \quad \text {and} \quad Z_s^{y,t} {:}=\sum _{x \in \mathbb {Z}^d} Z_{x,s}^{y,t}. \end{aligned}$$

Since $c(\beta )^{-(t-s+1)} \langle e^{\beta \mathcal {A}_s^t} \rangle = 1$ for every realization of $\gamma$, we have $\langle Z_{x,s}^t \rangle = \langle Z_s^{y,t} \rangle = 1$. Notice that the law of the stochastic process $(Z_{x,s}^{s+\tau })_{\tau \in \mathbb {N}_0}$ with respect to *Q* does not depend on *x* or *s*. Besides, $(Z_{x,s}^{s+\tau })_{\tau \in \mathbb {N}_0}$ and $(Z_{t-\tau }^{y,t})_{\tau \in \mathbb {N}_0}$ have the same law.

### Remark 1

This is essentially the model considered by Sinai, where *F*(*x*, *t*) in [3] corresponds to $\beta \xi (x,t)$ in our setting. Furthermore, the partition function $Z_{x,k}^{y,n}$ from [3] becomes $c(\beta )^{n-k+1} Z_{x,k}^{y,n}$ in our notation.

Given $z \in \mathbb {Z}^d$ and $s \in \mathbb {Z}$, define

$$\begin{aligned} h(z, s) {:}=\dfrac{e^{\beta \xi (z,s)} - c(\beta )}{c(\beta )}. \end{aligned}$$

As shown in the proof of Theorem 2 in [3], $Z_{x,s}^{y,t}$ can be written as

2 $$\begin{aligned} q_{t-s}^{y-x} + \sum _{r=1}^{t-s+1} \sum _{\begin{array}{c} s \le i_1< \cdots < i_r \le t, \\ z_1, \ldots , z_r \in \mathbb {Z}^d \end{array}} q_{i_1-s}^{z_1 - x} q_{i_2 - i_1}^{z_2 - z_1} \ldots q_{i_r - i_{r-1}}^{z_r - z_{r-1}} q_{t- i_r}^{y- z_r} \prod _{j=1}^r h(z_j, i_j). \end{aligned}$$

Similarly, one obtains the expansions

3 $$\begin{aligned} Z_s^{y,t} = 1 + \sum _{r=1}^{t-s+1} \sum _{\begin{array}{c} s \le i_1< \cdots < i_r \le t, \\ z_1, \ldots , z_r \in \mathbb {Z}^d \end{array}} q_{i_2 -i_1}^{z_2 - z_1} \ldots q_{t - i_r}^{y - z_r} \prod _{j=1}^r h(z_j, i_j) \end{aligned}$$

and

4 $$\begin{aligned} Z_{x,s}^{t} = 1 + \sum _{r=1}^{t-s+1} \sum _{\begin{array}{c} s \le i_1< \cdots < i_r \le t, \\ z_1, \ldots , z_r \in \mathbb {Z}^d \end{array}} q_{i_1-s}^{z_1 - x} \ldots q_{i_r - i_{r-1}}^{z_r - z_{r-1}} \prod _{j=1}^r h(z_j, i_j). \end{aligned}$$

### Convergence to Limiting Partition Functions

As in [3], define

5 $$\begin{aligned} \alpha _d {:}=\sum _{t=1}^{\infty } \sum _{z \in \mathbb {Z}^d} \left( q_t^z \right) ^2. \end{aligned}$$

It is well known that $q_t^z \lesssim t^{-\frac{d}{2}}$ (see, e.g., [11] or Lemma 7). Therefore, as $d \ge 3$, there is a constant $C > 0$ such that

$$\begin{aligned} \alpha _d \le C \sum _{t=1}^{\infty } \frac{1}{t^{\frac{d}{2}}} \sum _{z \in \mathbb {Z}^d} q_t^z = C\sum _{t=1}^{\infty } \frac{1}{t^{\frac{d}{2}}} < \infty . \end{aligned}$$

We also define

6 $$\begin{aligned} \lambda {:}=c(\beta )^{-2} c(2 \beta ) - 1. \end{aligned}$$

The following convergence statement for partition functions corresponds to Theorem 1 in [3].

#### Theorem 1

For $\beta$ so small that $\alpha _d \lambda < 1$, the following holds: As $t \rightarrow \infty$, $Z_{x,s}^t$ converges in $L^2(Q)$ to a limiting partition function $Z_{x,s}^{\infty }$.

#### Remark 2

Due to symmetry, we also have that

$$\begin{aligned} Z_{-\infty }^{y,t} := \lim _{s \rightarrow -\infty } Z_s^{y,t} \end{aligned}$$

exists in the sense of $L^2(Q)$ for all $y \in \mathbb {Z}^d$ and $t \in \mathbb {Z}$.

#### Remark 3

As pointed out by Bolthausen [2], $(Z_{x,s}^t)_{t \ge s}$ is a martingale with respect to the filtration $\mathcal {F}_t := \sigma (\xi (y,u): s \le u \le t, y \in \mathbb {Z}^d)$, so convergence to the limiting partition functions also holds *Q*-almost surely by the Martingale Convergence Theorem.

#### Proof of Theorem 1

We follow the approach in [3]. The right-hand side of (4) has an orthogonality structure which we will exploit. Since *h*(*z*, *s*) and $h(z',s')$ are independent if $z \ne z'$ or if $s \ne s'$, and since $\langle h(z,s) \rangle = 0$, we have with Jensen’s Inequality and Fubini’s Theorem that $\langle (Z^t_{x,s})^2 \rangle$ is bounded from above by

7 $$\begin{aligned} 2 + 2 \sum _{r=1}^{\infty } \sum _{\begin{array}{c} s \le i_1< \cdots < i_r, \\ z_1, \ldots , z_r \in \mathbb {Z}^d \end{array}} \left( q_{i_1-s}^{z_1-x} \right) ^2 \ldots \left( q_{i_r - i_{r-1}}^{z_r - z_{r-1}} \right) ^2 \biggl \langle \prod _{j=1}^r h(z_j, i_j)^2 \biggr \rangle . \end{aligned}$$

Since $\langle h(z,s)^2 \rangle = c(\beta )^{-2} c(2 \beta ) - 1$, we find

8 $$\begin{aligned} \biggl \langle \prod _{j=1}^r h(z_j, i_j)^2 \biggr \rangle = \prod _{j=1}^r \left( c(\beta )^{-2} c(2 \beta ) - 1 \right) = \lambda ^r. \end{aligned}$$

Since $\alpha _d \lambda < 1$, one has $\sum _{r=1}^{\infty } (\alpha _d \lambda )^r < \infty$, so the expression in (7) is finite. As a result,

$$\begin{aligned} \sup _{t > s} \left\langle \left( Z_{x,s}^t \right) ^2 \right\rangle < \infty , \end{aligned}$$

which yields $L^2$-convergence by the Martingale Convergence Theorem. $\square$

The following theorem gives a rate of convergence to the limiting partition function $Z_{x,s}^{\infty }$, which is needed to prove the factorization formula in Theorem 3.

#### Theorem 2

For $\beta$ so small that $\alpha _d \lambda < 1$ and for $\theta \in (0, \min \{\tfrac{d}{2}-1,-\ln (\alpha _d \lambda )\})$, one has

$$\begin{aligned} \lim _{t \rightarrow \infty } (t-s)^{\theta } \left\langle \left( Z_{x,s}^t - Z_{x,s}^{\infty } \right) ^2 \right\rangle = 0. \end{aligned}$$

#### Proof

For an integer $t \ge s$, let

$$\begin{aligned} M_t {:}=\left\langle \left( Z_{x,s}^t - 1 \right) ^2 \right\rangle = \sum _{r=1}^{t-s+1} \lambda ^r \sum _{\begin{array}{c} s \le i_1< \cdots < i_r \le t, \\ z_1, \ldots , z_r \in \mathbb {Z}^d \end{array}} \left( q_{i_1 - s}^{z_1 - x} \right) ^2 \ldots \left( q_{i_r - i_{r-1}}^{z_r - z_{r-1}} \right) ^2, \end{aligned}$$

which is monotone increasing in *t*. Set

$$\begin{aligned} M :=\lim _{t \rightarrow \infty } M_t \in (0,+\infty ]. \end{aligned}$$

Then, for $$t > s$$,

9 $$\begin{aligned}&\left\langle \left( Z_{x,s}^t - Z_{x,s}^{\infty } \right) ^2 \right\rangle = \lim _{T \rightarrow \infty } \left\langle \left( Z^t_{x,s} - Z^T_{x,s} \right) ^2 \right\rangle \le 2 (M - M_t) \end{aligned}$$

10 $$\begin{aligned}&\quad \le 2 \sum _{1 \le r \le \ln (t-s)} \lambda ^r \sum _{\begin{array}{c} s \le i_1< \cdots < i_r, i_r > t, \\ z_1, \ldots , z_r \in \mathbb {Z}^d \end{array}} \left( q_{i_1 - s}^{z_1 - x} \right) ^2 \ldots \left( q_{i_r - i_{r-1}}^{z_r - z_{r-1}} \right) ^2 \end{aligned}$$

11 $$\begin{aligned}&\qquad + 2 \sum _{r > \ln (t-s)} \lambda ^r \sum _{\begin{array}{c} s \le i_1< \cdots < i_r, \\ z_1, \ldots , z_r \in \mathbb {Z}^d \end{array}} \left( q_{i_1 - s}^{z_1 - x} \right) ^2 \ldots \left( q_{i_r - i_{r-1}}^{z_r - z_{r-1}} \right) ^2. \end{aligned}$$

The expression in (11) is dominated by

$$\begin{aligned} \sum _{r > \ln (t-s)} (\alpha _d \lambda )^r \lesssim (t-s)^{\ln (\alpha _d \lambda )}, \end{aligned}$$

and

$$\begin{aligned} \lim _{t \rightarrow \infty } (t-s)^{\theta } (t-s)^{\ln (\alpha _d \lambda )} = 0, \quad \theta \in (0, -\ln (\alpha _d \lambda )). \end{aligned}$$

The expression in (10) is dominated by

$$\begin{aligned}&\sum _{1 \le r \le \ln (t-s)} \lambda ^r \sum _{\begin{array}{c} t_1, \ldots , t_r \in \mathbb {N}, \\ t_1 + \cdots + t_r > t-s \end{array}} \sum _{x_1, \ldots , x_r \in \mathbb {Z}^d} \left( q_{t_1}^{x_1} \right) ^2 \ldots \left( q_{t_r}^{x_r} \right) ^2 \\&\quad \le \sum _{1 \le r \le \ln (t-s)} \lambda ^r \sum _{l=1}^r \sum _{\begin{array}{c} t_1, \ldots , t_r \in \mathbb {N}, \\ t_l \ge \frac{t-s}{\ln (t-s)} \end{array}} \prod _{k=1}^r \biggl (\sum _{x_k \in \mathbb {Z}^d} \left( q_{t_k}^{x_k} \right) ^2 \biggr ) \\&\quad \lesssim \sum _{r = 1}^{\infty } r (\alpha _d \lambda )^r \sum _{j \ge \frac{t-s}{\ln (t-s)}} \frac{1}{j^{\frac{d}{2}}} \lesssim \sum _{r=1}^{\infty } r (\alpha _d \lambda )^r \left( \frac{t-s}{\ln (t-s)} \right) ^{1 - \frac{d}{2}}, \end{aligned}$$

and

$$\begin{aligned} \lim _{t \rightarrow \infty } (t-s)^{\theta } \left( \frac{t-s}{\ln (t-s)} \right) ^{1-\frac{d}{2}} = 0, \quad \theta \in \left( 0, \tfrac{d}{2}-1\right) . \end{aligned}$$

$$\square $$

### Factorization Formula

The following factorization formula for the partition function $Z_{x,s}^{y,t}$ with fixed starting and endpoint is the main result of this article.

#### Theorem 3

Let $\beta$ be so small that $\alpha _d \lambda < 1$. For every $\sigma \in (0,1)$, no matter how close to 1, there exists $\theta = \theta (\sigma ) > 0$ such that for all $x,y\in \mathbb {Z}^d$ and $s < t$ with $\Vert x - y \Vert < (t - s)^\sigma$, the partition function $Z_{x,s}^{y,t}$ has the representation

12 $$\begin{aligned} Z_{x,s}^{y,t} = q_{t-s}^{y-x} \left( Z_{x,s}^{\infty } Z_{-\infty }^{y,t} + \delta _{x,s}^{y,t} \right) , \end{aligned}$$

where the error term $\delta _{x,s}^{y,t}$ defined by the formula above satisfies

13 $$\begin{aligned} \lim _{(t-s) \rightarrow \infty } (t-s)^{\theta } \sup _{x, y \in \mathbb {Z}^d: \Vert x-y\Vert < (t-s)^{\sigma }} \langle |\delta _{x,s}^{y,t} |\rangle = 0. \end{aligned}$$

Theorem 3 is proved in Sect. 4. Notice that the formula is similar to the ones obtained by Sinai in [3, Theorem 2] and Kifer in [6, Theorem 6.1]. However, we show that the error term is small not only within the diffusive regime $\Vert x-y\Vert < O(t-s)^{\frac{1}{2}}$, but also for $\Vert x-y\Vert < (t-s)^{\sigma }$ with $\sigma$ arbitrarily close to 1. This extension beyond the diffusive regime is nontrivial because the error term in (12) is multiplied by the random-walk transition probability $q_{t-s}^{y-x}$, which is itself extremely small for $\Vert x-y \Vert \ge (t-s)^{\frac{1}{2}}$. In a forthcoming publication, we rely heavily on a continuous-time version of Theorem 3 to prove a uniqueness statement for global solutions to the semi-discrete stochastic heat equation.

### Correlations for the Field of Limiting Partition Functions

As mentioned in Sect. 1, the distribution for the field of limiting partition functions $(Z_{x,s}^{\infty })_{x \in \mathbb {Z}^d, s \in \mathbb {Z}}$ is an interesting object to study, with several important questions still open. Below, we state asymptotics for the spatial and temporal correlations of this field.

#### Theorem 4

Let $\beta$ be so small that $\alpha _d \lambda < 1$. Then the spatial and temporal correlations for the field of limiting partition functions $(Z_{x,s}^{\infty })_{x \in \mathbb {Z}^d, s \in \mathbb {Z}}$ have the following asymptotics.

1. $$\qquad \displaystyle \lim _{\begin{array}{c} \Vert y\Vert \rightarrow \infty , \\ \Vert y\Vert _1 \equiv 0 \end{array}} \Vert y\Vert ^{d-2} \left( \langle Z_{0,0}^{\infty } Z_{y,0}^{\infty } \rangle - \langle Z_{0,0}^{\infty } \rangle \langle Z_{y,0}^{\infty } \rangle \right) \in (0,\infty )$$;
2. $$\qquad \displaystyle \lim _{\begin{array}{c} |s |\rightarrow \infty , \\ s \equiv 0 \end{array}} |s |^{\frac{d}{2} - 1} \left( \langle Z_{0,0}^{\infty } Z_{0,s}^{\infty } \rangle - \langle Z_{0,0}^{\infty } \rangle \langle Z_{0,s}^{\infty } \rangle \right) \in (0, \infty )$$.

It is necessary to take the limit in part (1) along sequences $(y_n)$ such that $\Vert y_n\Vert _1 \equiv 0$ for all *n*, as $Z_{0,0}^{\infty }$ and $Z_{y,0}^{\infty }$ are independent if $\Vert y\Vert _1 \equiv 1$. A similar observation applies to the limit in part (2). The proof of Theorem 4 relies on the following estimates for simple symmetric random walk on ${\mathbb {Z}}^d$, $d \ge 3$.

#### Lemma 5

The following statements hold:

1. $$\qquad \displaystyle \lim _{\begin{array}{c} \Vert y\Vert \rightarrow \infty , \\ \Vert y\Vert _1 \equiv 0 \end{array}} \Vert y\Vert ^{d-2} \sum _{t=0}^{\infty } \sum _{x \in \mathbb {Z}^d} q_t^x q_t^{y-x} \in (0,\infty )$$;
2. $$\qquad \displaystyle \lim _{\begin{array}{c} s \rightarrow \infty , \\ s \equiv 0 \end{array}} s^{\frac{d}{2}-1} \sum _{t=0}^{\infty } \sum _{x \in \mathbb {Z}^d} q_t^x q_{s+t}^x \in (0, \infty )$$.

#### Proof

For $y \in \mathbb {Z}^d$ whose 1-norm is even,

$$\begin{aligned} \sum _{t=0}^{\infty } \sum _{x \in \mathbb {Z}^d} q_t^x q_t^{y-x} = \sum _{t=0}^{\infty } q_{2t}^y = G(0,y), \end{aligned}$$

where *G* denotes the Green’s function for simple symmetric random walk on $\mathbb {Z}^d$. Theorem 4.3.1 in [11] implies that

$$\begin{aligned} \lim _{\begin{array}{c} \Vert y\Vert \rightarrow \infty , \\ \Vert y\Vert _1 \equiv 0 \end{array}} \Vert y\Vert ^{d-2} G(0,y) \in (0,\infty ), \end{aligned}$$

so (1) follows. To prove (2), first notice that for every even $s \in \mathbb {N}_0$,

$$\begin{aligned} \sum _{t=0}^{\infty } \sum _{x \in \mathbb {Z}^d} q_t^x q_{s+t}^x = \sum _{t=0}^{\infty } q_{s+2t}^0 = \sum _{t=s/2}^{\infty } q_{2t}^0. \end{aligned}$$

It is well known (see, e.g., [12, Chapter 1]) that

$$\begin{aligned} \lim _{t \rightarrow \infty } t^{\frac{d}{2}} q_{2t}^0 =: c \in (0, \infty ). \end{aligned}$$

Let $\epsilon > 0$. Then there exists $T \in \mathbb {N}$ such that

$$\begin{aligned} c - \epsilon \le t^{\frac{d}{2}} q_{2t}^0 \le c + \epsilon , \quad \forall t \ge T. \end{aligned}$$

Thus, for *s* even and $\ge 2T$,

$$\begin{aligned} \sum _{t = s/2}^{\infty } q_{2t}^0 \le (c+\epsilon ) \sum _{t = s/2}^{\infty } t^{-\frac{d}{2}} \le (c+\epsilon ) \frac{2}{d-2} \left( \frac{s}{2} - 1 \right) ^{1-\frac{d}{2}} \end{aligned}$$

and

$$\begin{aligned} \sum _{t = s/2}^{\infty } q_{2t}^0 \ge (c-\epsilon ) \frac{2}{d-2} \left( \frac{s}{2} \right) ^{1-\frac{d}{2}}. \end{aligned}$$

Hence,

$$\begin{aligned} \limsup _{\begin{array}{c} s \rightarrow \infty , \\ s \equiv 0 \end{array}} s^{\frac{d}{2}-1} \sum _{t = s/2}^{\infty } q_{2t}^0 \le (c+ \epsilon ) \frac{2^{\frac{d}{2}}}{d-2} \quad \text {and} \quad \liminf _{\begin{array}{c} s \rightarrow \infty , \\ s \equiv 0 \end{array}} s^{\frac{d}{2}-1} \sum _{t = s/2}^{\infty } q_{2t}^0 \ge (c-\epsilon ) \frac{2^{\frac{d}{2}}}{d-2}. \end{aligned}$$

Since $\epsilon$ was arbitrarily chosen, we obtain (2). $\square$

#### Proof of Theorem 4

For $y \in \mathbb {Z}^d$ such that $\Vert y\Vert _1 \equiv 0$ and $t \in \mathbb {N}$, the expansion in (4) along with the properties of *h*(*z*, *s*) yield

$$\begin{aligned} \langle Z_{0,0}^t Z_{y,0}^t \rangle = 1 + \sum _{r=1}^{t+1} \lambda ^r \sum _{\begin{array}{c} 0 \le i_1< \cdots < i_r \le t, \\ z_1, \ldots , z_r \in \mathbb {Z}^d \end{array}} q_{i_1}^{z_1} q_{i_1}^{z_1 - y} \left( q_{i_2 - i_1}^{z_2 - z_1} \right) ^2 \ldots \left( q_{i_r - i_{r-1}}^{z_r - z_{r-1}} \right) ^2. \end{aligned}$$

Along the lines of the proof of Theorem 2, one can easily show that, as $t \rightarrow \infty$, the expression on the right-hand side converges to

$$\begin{aligned} 1 + \sum _{r=1}^{\infty } \lambda ^r \sum _{\begin{array}{c} 0 \le i_1< \cdots < i_r, \\ z_1, \ldots , z_r \in \mathbb {Z}^d \end{array}} q_{i_1}^{z_1} q_{i_1}^{z_1 - y} \left( q_{i_2-i_1}^{z_2-z_1} \right) ^2 \ldots \left( q_{i_r - i_{r-1}}^{z_r - z_{r-1}} \right) ^2. \end{aligned}$$

Therefore,

$$\begin{aligned} \langle Z_{0,0}^{\infty } Z_{y,0}^{\infty } \rangle - \langle Z_{0,0}^{\infty } \rangle \langle Z_{y,0}^{\infty } \rangle&= \sum _{r=1}^{\infty } \lambda ^r \sum _{\begin{array}{c} 0 \le i_1< \cdots < i_r, \\ z_1, \ldots , z_r \in \mathbb {Z}^d \end{array}} q_{i_1}^{z_1} q_{i_1}^{z_1 - y} \left( q_{i_2 - i_1}^{z_2 - z_1} \right) ^2 \ldots \left( q_{i_r -i_{r-1}}^{z_r - z_{r-1}} \right) ^2 \\&= \sum _{i=0}^{\infty } \sum _{x \in \mathbb {Z}^d} q_i^x q_i^{x-y} \alpha _d^{-1} \sum _{r=1}^{\infty } (\alpha _d \lambda )^r, \end{aligned}$$

and part (1 ) follows from Lemma 5. The proof of part (2) is similar and we omit it. $\square$

## Transition Probabilities for the Simple Symmetric Random Walk

In this section we collect several estimates on transition probabilities for the discrete-time simple symmetric random walk on $\mathbb {Z}^d$, some of which are presumably well known to specialists. Appendix A will be devoted to the proofs of the results presented here.

Let $(\gamma _n)_{n \in \mathbb {N}_0}$ be a discrete-time simple symmetric random walk on $\mathbb {Z}^d$ starting at the origin.

### Lemma 6

There exist constants $c_1, c_2 > 0$ such that the following holds: For every $\sigma \in (\tfrac{3}{4},1)$ and ${\tilde{\sigma }} \in (\sigma , 1)$, there exists $T \in \mathbb {N}$ such that for every $t \ge T$ and $y \in \mathbb {Z}^d$ with $q^y_t > 0$ and $\Vert y\Vert \le t^{\sigma }$,

14 $$\begin{aligned} q^y_t \ge c_1 \left( \tfrac{d}{2 \pi t} \right) ^{\frac{d}{2}} \exp \big ( -\tfrac{d}{2t} \Vert y\Vert ^2 \big ) \exp \Big (-c_2 t^{4 {{\tilde{\sigma }}} - 3} \Big ). \end{aligned}$$

### Lemma 7

There exists $c_1 > 0$ such that for every $y \in \mathbb {Z}^d$ and for every linear functional $\varphi$ on $\mathbb {R}^d$ with $|\varphi (x) |\le \Vert x\Vert$, $x \in \mathbb {R}^d$, we have

$$\begin{aligned} q_t^y e^{\varphi (y)} \le c_1 t^{-\frac{d}{2}} \sum _{z \in \mathbb {Z}^d} q_t^z e^{\varphi (z)}, \quad \forall t \in \mathbb {N}. \end{aligned}$$

In particular,

$$\begin{aligned} q_t^y \lesssim t^{-\frac{d}{2}}. \end{aligned}$$

Fix a linear functional $\varphi$ on $\mathbb {R}^d$ such that $|\varphi (x) |\le \Vert x\Vert$ for every $x \in \mathbb {R}^d$. To simplify notation, we set $\varphi _j {:}=\varphi (e_j)$ for $1 \le j \le d$, where $\{ e_j \}$ is the standard basis in $\mathbb {R}^d$. Define for every $\theta = (\theta ^1, \ldots , \theta ^d) \in \mathbb {R}^d$

15 $$\begin{aligned} \Phi (\theta ) {:}=\textbf{E}\left[ e^{i \theta \cdot \gamma _1} e^{\varphi (\gamma _1)} \right] = \frac{1}{2d} \sum _{j=1}^d \left( e^{i \theta ^j} e^{\varphi _j} + e^{-i \theta ^j} e^{-\varphi _j} \right) , \end{aligned}$$

where *i* is the imaginary unit. Notice that for every $\theta \in \mathbb {R}^d$,

16 $$\begin{aligned} \big |\Phi (\theta ) \big |\le \Phi (0) = \sum _{z \in \mathbb {Z}^d} q_1^z e^{\varphi (z)}, \end{aligned}$$

where 0 is the zero vector in $\mathbb {R}^d$. Furthermore,

17 $$\begin{aligned} \Phi (0)^t = \sum _{y \in \mathbb {Z}^d} q_t^y e^{\varphi (y)}, \quad \forall t \in \mathbb {N}_0. \end{aligned}$$

Notice also that $\Phi$ is $2\pi$-periodic in every argument, so it will be convenient to work with the cube $\mathcal {C}{:}=(-\tfrac{\pi }{2}, \tfrac{3 \pi }{2}]^d$. It is not hard to see that the inequality (16) is strict for all $\theta \in \mathcal {C}$ except for $\theta ^0 {:}=(0, \ldots , 0)$ and $\theta ^1 {:}=(\pi , \ldots , \pi )$.

### Lemma 8

There exist $\rho _1, \rho _2 > 0$ such that the following holds: For every $t \in \mathbb {N}$ and for every $z \in \mathbb {Z}^d$ such that $\Vert z\Vert \le \rho _1 t$ and $q^z_t > 0$, there exists a linear functional $\varphi$ on $\mathbb {R}^d$ of norm $\Vert \varphi \Vert \le \rho _2 \tfrac{\Vert z\Vert }{t}$ which satisfies

$$\begin{aligned} \frac{1}{(2 \pi )^d} \int _{\mathcal {C}} \big |\Phi (\theta ) \big |^t \, d \theta \le \left( 1 + O(t^{-\frac{2}{5}}) \right) q^z_t e^{\varphi (z)} \end{aligned}$$

and

$$\begin{aligned} q_t^z e^{\varphi (z)} \gtrsim t^{-\frac{d}{2}} \sum _{y \in \mathbb {Z}^d} q_t^y e^{\varphi (y)}. \end{aligned}$$

### Lemma 9

There exist constants $\rho , c > 0$ such that for every $t, t' \in \mathbb {N}$ and $z, z' \in \mathbb {Z}^d$ with $\Vert z\Vert \le \rho t$ and $q^z_t > 0$, one has

$$\begin{aligned} \frac{q_{t'}^{z'}}{q_t^z} \le \left( 1+O(t^{-\frac{2}{5}}) \right) \exp \left( c \left( \frac{\Vert z\Vert }{t} \left( \Vert z-z'\Vert + |t' - t |\right) + \ln (t) \frac{|t-t' |}{t} \right) \right) . \end{aligned}$$

## Proof of Theorem 3

The main idea behind the factorization formula, which goes back at least to [3], is that there is strong averaging for times neither too close to *s* nor too close to *t*.

Consider the representation of $Z_{x,s}^{y,t}$ in (2). For fixed *r*, $i_1, \ldots , i_r$, and $z_1, \ldots , z_r$, the random walk is pinned to the points $z_1, \ldots , z_r$ at the corresponding times $i_1, \ldots , i_r$. The proof of Theorem 1 suggests that the contribution to $Z_{x,s}^{y,t}$ from *r* on the order of $(t-s)$ is negligible. If *r* is not on the order of $(t-s)$, at least one of the gaps $i_j - i_{j-1}$ must be in some sense large (see Sect. 4.1). In Sect. 4.2.2, we show that the contribution to $Z_{x,s}^{y,t}$ coming from two or more large gaps is negligible as well. Thus, the main contribution comes from having exactly one large gap $i_j - i_{j-1}$, which is then on the order of $(t-s)$. In order for $q_{i_j - i_{j-1}}^{z_j - z_{j-1}}$ to be positive, $z_{j-1}$ must be close to *x* and $z_j$ must be close to *y*. The transition probability $q_{i_j - i_{j-1}}^{z_j - z_{j-1}}$ is then close to $q_{t-s}^{y-x}$.

Notice that to prove Theorem 3, it is enough to show that if $\alpha _d \lambda < 1$, then for every $\sigma \in (0,1)$ there exists $\theta > 0$ such that

$$\begin{aligned} \lim _{t \rightarrow \infty } t^{\theta } \sup _{y \in \mathbb {Z}^d: \Vert y\Vert < t^{\sigma }} \langle |\delta _{0,0}^{y,t} |\rangle = 0. \end{aligned}$$

This is because for a fixed realization $\omega$ of the disorder, $\delta _{x,s}^{y,t}(\omega )$ can be written as $\delta _{0,0}^{y-x,t-s}({{\hat{\omega }}})$, where ${{\hat{\omega }}}$ is obtained by shifting $\omega$ in space and time. The distribution of the disorder is invariant under such shifts.

For $t \in \mathbb {N}_0$ and $r \in \{1, \ldots , t+1\}$, let

$$\begin{aligned} I(t,r) {:}=\{\textbf{i}= (i_1,\ldots ,i_r) \in \mathbb {N}_0^r \,:\, 0 \le i_1< \cdots < i_r \le t\}. \end{aligned}$$

For $\textbf{i}\in I(t,r)$ and $\textbf{z}= (z_1, \ldots , z_r) \in (\mathbb {Z}^d)^r$, define

$$\begin{aligned} q_t^y(\textbf{i},\textbf{z}) {:}=q_{i_1}^{z_1} q_{i_2 - i_1}^{z_2 - z_1} \ldots q_{t - i_r}^{y-z_r}. \end{aligned}$$

With this notation, the expansion in (2) becomes

18 $$\begin{aligned} Z_{0,0}^{y,t} = q_{t}^{y} + \sum _{r=1}^{t+1} \sum _{\textbf{i}\in I(t,r), \textbf{z}} q_{t}^y(\textbf{i},\textbf{z}) \prod _{j=1}^r h(z_j, i_j), \end{aligned}$$

where one should recall from Sect. 1 the notational shorthand $\sum _{\textbf{z}}$ for summation over all $\textbf{z}= (z_1, \ldots , z_r) \in (\mathbb {Z}^d)^r$. The first step is to split the double sum into terms according to the size of the largest gap between indices, as discussed in Sect. 4.1.

### Large and Huge Gaps

If there exist $\sigma \in (0,1)$ and $\theta > 0$ such that (13) (the convergence of the error term in the factorization formula) holds, then (13) also holds for ${{\tilde{\sigma }}}$ and $\theta$, where ${{\tilde{\sigma }}}$ can be any value in $(0, \sigma )$. There is then no loss of generality in assuming that $\sigma > 3/4$, and one may even think of $\sigma$ as being very close to 1. For a collection of indices $0 ={:}i_0 \le i_1< \cdots < i_r \le i_{r+1} {:}=t$, the *gaps* are the differences between consecutive indices, i.e., $i_1 - i_0, i_2 - i_1, \ldots , i_{r+1} - i_r$. To quantify what it means to have many gaps, we fix positive constants $\kappa _1$, $\kappa _2 \in (\tfrac{1}{2} (3 \sigma - 1), \sigma )$ such that $\kappa _1 < \kappa _2$. Let $T_{\kappa _2} \in \mathbb {N}$ be so large that $2 (t - t^{\kappa _2}) > t$ for all $t \ge T_{\kappa _2}$. Then define

$$\begin{aligned} k(t) {:}={\left\{ \begin{array}{ll} (t-T_{\kappa _2} )^{\kappa _1} - 1, &{} \quad (t-T_{\kappa _2} )^{\kappa _1} - 1 \ge 1, \\ 0, &{} \quad (t-T_{\kappa _2})^{\kappa _1} - 1 < 1. \end{array}\right. } \end{aligned}$$

Note that *k*(*t*) grows with *t* like $t^{\kappa _1}$. We say that a collection of indices $0 \le i_1< \cdots < i_r \le t$ has *many gaps* if $r > k(t)$.

To classify the size of a gap between indices, fix another constant $\xi$ such that $0< \xi < \min \big \{ 1-\sigma , \kappa _2 - \kappa _1 \}$. One should think of $\xi$ as being very close to 0. Note that $\xi + \kappa _1 < 1$ and that $\xi < \kappa _1$, the latter because of $\xi< 1 - \sigma< 1/4< \tfrac{1}{2} (\tfrac{9}{4} - 1) < \kappa _1$. Let $t \in \mathbb {N}$ such that $k(t) \ge 1$, *r* such that $1 \le r \le k(t)$, and consider a sequence of indices $0 = i_0 \le i_1< \cdots < i_r \le i_{r+1} = t$. We say that the gap between two consecutive indices $i_{j-1}$ and $i_j$ is

- *large* if $$i_j - i_{j-1} \ge t^{\xi }$$;
- *huge* if $$i_j - i_{j-1} \ge t - r t^{\xi }$$.

Observe that the size of the largest gap is necessarily greater than $t / (r+1) \ge t^{1-\kappa _1} \ge t^{\xi }$, so there is at least one large gap. A huge gap is necessarily large. If there is only one large gap, then all other gaps are of size less than $t^{\xi }$, so this large gap is even huge. Thus, if there is no huge gap, there are at least two large ones. Since *t* must be greater than $T_{\kappa _2}$ in order for $k (t) \ge 1$ to hold, we have $2(t-r t^{\xi })> 2 (t-t^{\kappa _1+\xi })> 2 (t-t^{\kappa _2}) > t,$ so there can be at most one huge gap. Note, however, that a huge gap is not necessarily the only large one.

Let us introduce some more notation. Fix $r \in \mathbb {N}$ and $t \in \mathbb {N}_0$. For $m \in \mathbb {N}$ such that $1 \le m \le r+1$, define the following set of *r*-tuples:

$$\begin{aligned} I_1 (t, r, m) {:}=\left\{ (i_1, \ldots , i_r) \in I(t,r) ~:~ \text {the gap between }i_{m-1}\hbox { and }i_m\hbox { is huge} \right\} . \end{aligned}$$

Also define

$$\begin{aligned} I_2 (t, r) {:}=\left\{ (i_1, \ldots , i_r) \in I(t,r) ~:~ \text {there is no huge gap} \right\} . \end{aligned}$$

For *t* so large that $k(t) \ge 1$, we decompose the expansion of $Z^{y,t}_{0,0}$ in (18) as follows:

$$\begin{aligned} Z_{0,0}^{y,t} = q_t^y + \sum _{j=1}^3 B_j^{y,t}, \end{aligned}$$

where,

$$\begin{aligned} B_1^{y,t} {:}=&\sum _{k(t) < r \le t +1} \sum _{\textbf{i}\in I(t,r), \textbf{z}} q_{t}^y(\textbf{i},\textbf{z}) \prod _{j=1}^r h(z_j, i_j), \\ B_2^{y,t} {:}=&\sum _{1 \le r \le k(t)} \sum _{\textbf{i}\in I_2 (t, r), \textbf{z}} q_{t}^y(\textbf{i},\textbf{z}) \prod _{j=1}^r h(z_j, i_j), \\ B_3^{y,t} {:}=&\sum _{1 \le r \le k(t)} \sum _{m=1}^{r+1} \sum _{\textbf{i}\in I_1 (t, r, m), \textbf{z}} q_{t}^y(\textbf{i},\textbf{z}) \prod _{j=1}^r h(z_j, i_j). \end{aligned}$$

With this decomposition in hand, Theorem 3 follows immediately from the following lemma.

#### Lemma 10

(Central Lemma) Let $\beta >0$ be so small that $\alpha _d \lambda < 1$, and let $\sigma \in (0,1)$.

1. For every $$\theta > 0$$,
  19 $$\begin{aligned} \lim _{t \rightarrow \infty } t^{\theta } \sup _{y: \Vert y\Vert \le t^{\sigma }, q^y_t > 0} \frac{\langle |B_1^{y,t} |\rangle }{q_t^y} = 0. \end{aligned}$$
2. There exists $\theta > 0$ such that
  20 $$\begin{aligned} \lim _{t \rightarrow \infty } t^{\theta } \sup _{y: \Vert y\Vert \le t^{\sigma }, q^y_t > 0} \frac{\langle |B^{y,t}_2 |\rangle }{q_t^y} = 0. \end{aligned}$$
3. There exists $\theta > 0$ such that
  21 $$\begin{aligned} \lim _{t \rightarrow \infty } t^{\theta } \sup _{y: \Vert y\Vert \le t^{\sigma }, q_t^y > 0} \left\langle \left|1 + \frac{B_3^{y,t}}{q_t^y} - Z_{0,0}^{\infty } Z_{-\infty }^{y,t} \right|\right\rangle = 0. \end{aligned}$$

Sections 4.2 and 4.3 are devoted to the proof of this lemma.

### Proof of the Central Lemma, Parts 1 and 2: Small Contributions

In this section, we show that the contributions of the terms $B_1^{y,t}$ and $B_2^{y,t}$ to $Z_{0,0}^{y,t}$ are negligible. We start with the observation that, by Jensen’s inequality,

22 $$\begin{aligned} \left( \frac{1}{q^y_t} \Big \langle \big |B_j^{y,t} \big |\Big \rangle \right) ^2 \le \frac{1}{(q^y_t)^2} \left\langle \left( B_j^{y,t} \right) ^2 \right\rangle , \quad j = 1,2. \end{aligned}$$

#### Proof of Part 1: Many Gaps

Let $t \in \mathbb {N}$ be so large that $k(t) \ge 1$. Since $\alpha _d \lambda < 1$, (8) and the definition (5) of $\alpha _d$ let us estimate $\langle (B_1^{y,t})^2 \rangle$ as follows:

$$\begin{aligned} \left\langle \left( B_1^{y,t} \right) ^2 \right\rangle =&\sum _{k(t)< r \le t+1} \lambda ^r \sum _{\textbf{i}\in I(t,r), \textbf{z}} q^y_t(\textbf{i}, \textbf{z})^2 \\ \lesssim&\sum _{k(t) < r \le t + 1} (\alpha _d \lambda )^r \le \sum _{r > k(t)} (\alpha _d \lambda )^r \le \frac{(\alpha _d \lambda )^{k(t)}}{1 - \alpha _d \lambda }. \end{aligned}$$

Recall our assumption that $\sigma > \tfrac{3}{4}$. To estimate $1/(q_t^y)^2$ on the right-hand side of (22), fix ${\tilde{\sigma }} \in (\sigma , 1)$ such that $4 {\tilde{\sigma }} - 3 < 2 \sigma - 1$. By Lemma 6, there exist constants $c_1, c_2 > 0$ (independent of $\sigma , {\tilde{\sigma }}$) and $T \in \mathbb {N}$ (depending on $\sigma ,{\tilde{\sigma }}$) such that for every integer $t \ge T$ and $y \in \mathbb {Z}^d$ with $q_t^y > 0$ and $\Vert y \Vert \le t^{\sigma }$,

$$\begin{aligned} q^y_t&\ge c_1 \left( \tfrac{d}{2 \pi t} \right) ^{d/2} \exp \big ( -\tfrac{d}{2t} \Vert y\Vert ^2 \big ) \exp \left( -c_2 t^{4 {\tilde{\sigma }} - 3} \right) \\&\gtrsim t^{-d/2} \exp \Big ( - \tfrac{d}{2} t^{2 \sigma - 1} - c_2 t^{4 {\tilde{\sigma }} - 3} \Big ) \ge t^{-d/2} \exp \big ( - c t^{2 \sigma - 1} \big ) \end{aligned}$$

for some constant $c > 0$. Therefore,

$$\begin{aligned} \sup _{y: \Vert y\Vert \le t^{\sigma }, q^y_t > 0} \frac{1}{(q^y_t)^2} \left\langle \left( B_1^{y,t} \right) ^2 \right\rangle \lesssim t^d \ (\alpha _d \lambda )^{k(t)} \ \exp \big ( 2 c t^{2 \sigma - 1} \big ). \end{aligned}$$

Since $\kappa _1> \tfrac{1}{2} (3 \sigma -1) > 2 \sigma -1$, we have $t^{2 \sigma -1} / k(t) \rightarrow 0$ as $t \rightarrow \infty$, and therefore, for all $\theta > 0$,

$$\begin{aligned} t^{\theta } \sup _{y: \Vert y\Vert \le t^{\sigma }, q^y_t > 0} \frac{1}{(q^y_t)^2} \left\langle \left( B^{y,t}_1 \right) ^2 \right\rangle \lesssim t^{\theta +d} \ (\alpha _d \lambda )^{k(t)} \ \exp \big ( 2 c t^{2 \sigma - 1} \big ) \xrightarrow [t \rightarrow \infty ]{} 0. \end{aligned}$$

#### Proof of Part 2: No Huge Gaps

Let $t \in \mathbb {N}$ be so large that $k(t) \ge 1$. Then

23 $$\begin{aligned} \left\langle \left( B_2^{y,t} \right) ^2 \right\rangle = \sum _{1 \le r \le k(t)} \lambda ^r \sum _{\textbf{i}\in I_2(t,r), \textbf{z}} q^y_t(\textbf{i}, \textbf{z})^2 \lesssim \sum _{1 \le r \le k(t)} \lambda ^r M_{t,r}(y), \end{aligned}$$

where

$$\begin{aligned} M_{t,r}(y) {:}=\sum _{\begin{array}{c} \textbf{i}\in I_2(t,r), \textbf{z}\\ i_1 \ne 0, i_r \ne t \end{array}} q^y_t(\textbf{i}, \textbf{z})^2. \end{aligned}$$

Now we estimate $M_{t,r}(y)$. Let $r \in \mathbb {N}$ such that $1 \le r \le k(t)$, and $y \in \mathbb {Z}^d$ such that $\Vert y \Vert \le t^{\sigma }$ and $q^y_t > 0$. Given $\textbf{i}= (i_1, \ldots , i_r) \in I_2(t,r)$ such that $i_1 \ne 0$ and $i_r \ne t$, set $t_1 {:}=i_1, t_2 {:}=i_2 - i_1, \ldots , t_r {:}=i_r - i_{r-1}, t_{r+1} {:}=t - i_r$. And given $\textbf{z}= (z_1, \ldots , z_r) \in (\mathbb {Z}^d)^r$, set $x_1 {:}=z_1, x_2 {:}=z_2 - z_1, \ldots , x_r {:}=z_r - z_{r-1}, x_{r+1} {:}=y - z_r$. This change of variables yields

24 $$\begin{aligned} q_t^y(\textbf{i}, \textbf{z})^2 = \left( q_{t_1}^{x_1} \right) ^2 \ldots \left( q_{t_{r+1}}^{x_{r+1}} \right) ^2. \end{aligned}$$

For $\textbf{i}\in I_2(t,r)$, there is no huge gap and hence there are at least two large ones. Let

$$\begin{aligned} l {:}=\left|\left\{ 1 \le j \le r+1: \ t_j \ge t^{\xi } \right\} \right|- 1, \end{aligned}$$

i.e., $(l+1)$ gives the number of large gaps in $\textbf{i}$. There are $(r+1)$ possible slots for the largest gap (which is then also a *large gap*), and $$\left( {\begin{array}{c}r\\ l\end{array}}\right) $$ possible slots for the other *l* large gaps once the largest gap has been fixed. Together with (24), this yields the estimate

25 $$\begin{aligned} M_{t,r}(y) \le (r+1) \sum _{l=1}^r \left( {\begin{array}{c}r\\ l\end{array}}\right) M_{t,r,l}(y), \end{aligned}$$

where

$$\begin{aligned} M_{t,r,l}(y) := \qquad \sum _{{\begin{array}{c} t_1 + \cdots + t_{r+1} = t \\ x_1 + \cdots + x_{r+1} = y \\ t_{r+1} \ge t_1, \ldots , t_l \ge t^{\xi } \\ t_{l+1}, \ldots , t_r < t^{\xi } \end{array}}} \qquad \left( q_{t_1}^{x_1} \right) ^2 \ldots \left( q_{t_{r+1}}^{x_{r+1}} \right) ^2. \end{aligned}$$

The sum on the right-hand side is taken over all $t_1, \ldots , t_{r+1} \in \mathbb {N}$ and $x_1, \ldots , x_{r+1} \in \mathbb {Z}^d$ that satisfy the four conditions under the summation sign. In the special case $l=r$, the fourth condition is void.

For given positive integers $t_{l+1}, \ldots , t_r$ strictly less than $t^{\xi }$, set

$$\begin{aligned} t'(t_{l+1}, \ldots , t_r) = t' {:}=t - (t_{l+1} + \cdots + t_{r}); \end{aligned}$$

and for $x_{l+1}, \ldots , x_r \in \mathbb {Z}^d$, set

$$\begin{aligned} x'(x_{l+1}, \ldots , x_r) = x' {:}=y - (x_{l+1} + \cdots + x_{r}). \end{aligned}$$

If $l < r$, this lets us write

26 $$\begin{aligned} M_{t,r,l} (y)&= \quad \sum _{{\begin{array}{c} t_{l+1}, \ldots , t_{r} < t^\xi \end{array}}} \;\; \big ( q_{t_{l+1}}^{x_{l+1}} \big )^2 \cdots \big ( q_{t_r}^{x_r} \big )^2 M_{t,r,l}^{t'} (x'), \end{aligned}$$

27 $$\begin{aligned} \text {where} \quad M_{t,r,l}^{t'} (x')&{:}=\qquad \sum _{{ \begin{array}{c} t_1 + \cdots + t_l + t_{r+1} = t' \\ x_1 + \cdots + x_l + x_{r+1} = x' \\ t_{r+1} \ge t_1, \ldots , t_l \ge t^\xi \end{array}}} \qquad \big ( q_{t_1}^{x_1} \big )^2 \cdots \big ( q_{t_l}^{x_l} \big )^2 \big ( q_{t_{r+1}}^{x_{r+1}} \big )^2. \end{aligned}$$

We now search for a bound for $M_{t,r,l}^{t'} (x')$ when *t* is sufficiently large.

##### Claim 4.1

There exist constants $C, C', T > 0$ such that for every integer $t \ge T$ and for all $r, l, t', x'$ as above,

$$\begin{aligned} M_{t,r,l}^{t'} (x') \lesssim t^{- \xi /4} \ (q_t^y)^2 \ C^l \ t^{- \xi l (2d-5) / 4} \ \exp \Big ( C' (r-l) t^{\sigma + \xi - 1} \Big ), \end{aligned}$$

and, in the special case $l=r$,

$$\begin{aligned} M_{t,r,r}(y) \lesssim t^{- \xi /4} \ (q_t^y)^2 \ C^r \ t^{-\xi r (2d-5)/4}. \end{aligned}$$

We use Claim 4.1 to estimate $M_{t,r,l} (y)$ from (26) as follows:

$$\begin{aligned} M_{t,r,l} (y)&\lesssim \alpha _d^{r-l} \ t^{- \xi /4} \ (q_t^y)^2 \ C^l \ t^{- \xi l (2d-5) / 4} \ \exp \Big ( C' (r-l) t^{\sigma + \xi - 1} \Big ) \\&\le t^{- \xi /4} \ (q_t^y)^2 \ \Big ( C \ t^{- \xi (2d-5) / 4} \Big )^l \ \Big ( \alpha _d \exp \big ( C' t^{\sigma + \xi - 1} \big ) \Big )^{r-l}. \end{aligned}$$

Then we combine the above estimate with (25) to obtain

$$\begin{aligned} M_{t,r} (y)&\lesssim t^{- \xi /4} (q_t^y)^2 (r + 1) \sum _{l=1}^r \left( {\begin{array}{c}r\\ l\end{array}}\right) \Big ( C t^{- \xi (2d-5) / 4} \Big )^l \Big ( \alpha _d \exp \big ( C' t^{\sigma + \xi - 1} \big ) \Big )^{r-l} \\&\le t^{- \xi /4} \ (q_t^y)^2 \ (r+1) \ \Big ( C t^{- \xi (2d-5) / 4} + \alpha _d \exp \big ( C' t^{\sigma + \xi - 1} \big ) \Big )^r. \end{aligned}$$

Finally, combining this estimate with (22) and (23), we obtain

$$\begin{aligned} \left( \frac{1}{q^y_t} \left\langle \left|B^{y,t}_2 \right|\right\rangle \right) ^2 \lesssim t^{-\xi /4} \ \sum _{r = 1}^{\infty } \ (r + 1) \ \lambda ^r \ \Big ( C t^{- \xi (2d-5) / 4} + \alpha _d \exp \big ( C' t^{\sigma + \xi - 1} \big ) \Big )^r. \end{aligned}$$

Since $d \ge 3$ and since $\xi < 1- \sigma$, one has

$$\begin{aligned} \lim _{t \rightarrow \infty } t^{\theta } \sup _{y: \Vert y\Vert \le t^{\sigma }, q^y_t > 0} \left( \frac{1}{q^y_t} \left\langle \left|B^{y,t}_2 \right|\right\rangle \right) ^2 = 0 \end{aligned}$$

as long as $\theta < \xi / 4$ and hence (20) for every $\theta < \xi / 8$. To complete the proof of Part 2, it remains to prove Claim 4.1.

##### Proof of Claim 4.1

By Lemma 8, there exist constants $\rho _1, \rho _2 > 0$ such that for every $t \in \mathbb {N}$ and $y \in \mathbb {Z}^d$ with $\Vert y \Vert \le \rho _1 t$ and $q^y_t > 0$, there exists a linear functional $\varphi$ on $\mathbb {R}^d$ of norm $\Vert \varphi \Vert \le \rho _2 \Vert y \Vert / t$ which satisfies

28 $$\begin{aligned} q_t^y e^{\varphi (y)} \gtrsim t^{-d/2} \sum _{z \in \mathbb {Z}^d} q_t^z e^{\varphi (z)}. \end{aligned}$$

Fix $t \in \mathbb {N}$ so large that $k(t) \ge 1$ as well as $t^{\sigma } \le \rho _1 t$ and $\rho _2 t^{\sigma - 1} \le 1$. Let $y \in \mathbb {Z}^d$ such that $\Vert y \Vert \le t^{\sigma }$ and $q^y_t > 0$. The conditions $\rho _2 t^{\sigma - 1} \le 1$ and $\Vert y \Vert \le t^{\sigma }$ imply in particular that $\Vert \varphi \Vert \le 1$ for the linear function $\varphi$ corresponding to *t* and *y*. Let $t_1, \ldots , t_l, t_{r+1} \in \mathbb {N}$ and $x_1, \ldots , x_l, x_{r+1} \in \mathbb {Z}^d$ such that the conditions under the summation sign in (27) hold. In the special case $l=r$, replace $t'$ and $x'$ with *t* and *y*, respectively, here and in the remainder of the proof. By Lemma 7, there exists a constant $c_1 > 0$ such that

$$\begin{aligned} q_{t_1}^{x_1} \cdots q_{t_l}^{x_l} q_{t_{r+1}}^{x_{r+1}}&= e^{\varphi (-x')} \prod _{j \in \{1, \ldots , l, r+1\}} e^{\varphi (x_j)} q_{t_j}^{x_j} \\&\le e^{\varphi (-x')} \prod _{j \in \{1, \ldots , l, r+1\}} \biggl ( c_1 t_j^{-d/2} \sum _{z \in \mathbb {Z}^d} q_{t_j}^z e^{\varphi (z)} \biggr ) \\&= e^{\varphi (-x')} \sum _{z \in \mathbb {Z}^d} q_{t'}^z e^{\varphi (z)} \prod _{j \in \{1, \ldots , l, r+1\}} \biggl ( c_1 t_j^{-d/2} \biggr ) \\&= e^{\varphi (-x')} \ \Phi (0)^{t'} \prod _{j \in \{1, \ldots , l, r+1\}} \biggl ( c_1 t_j^{-d/2} \biggr ), \end{aligned}$$

where in the third line we used the fact that $\varphi$ is a linear functional, and in the fourth line we used (17), where $\Phi$ was defined in (15). Since $t' < t$ and $\Phi (0) \ge 1$, it follows from (28) that $\Phi (0)^{t'} \le \Phi (0)^t \lesssim t^{d/2} q_t^y e^{\varphi (y)}$. As a result, for all positive integers $t_1, \ldots , t_l, t_{r+1}$ such that $t_1 + \cdots + t_l + t_{r+1} = t'$ and $t_{r+1} \ge t_1, \ldots , t_l \ge t^{\xi }$, one has

$$\begin{aligned} \max _{x_1 + \cdots + x_l + x_{r+1} = x'} q_{t_1}^{x_1} \cdots q_{t_l}^{x_l} q_{t_{r+1}}^{x_{r+1}} \lesssim t^{d/2} \ q_t^y \ e^{\varphi (y - x')} \prod _{j \in \{1, \ldots , l, r+1\}} \biggl (c_1 t_j^{-d/2} \biggr ). \end{aligned}$$

Furthermore, the sum $\sum q_{t_1}^{x_1} \cdots q_{t_l}^{x_l} q_{t_{r+1}}^{x_{r+1}}$ over all tuples $(x_1, \ldots , x_l, x_{r+1})$ such that $x_1 + \cdots x_l + x_{r+1} = x'$ equals $q_{t'}^{x'}$, and by Lemma 9 there exist constants $c, \rho > 0$ such that

$$\begin{aligned} q_{t'}^{x'} \le q_t^y \big (1 + O(t^{-2/5}) \big ) \exp \Bigg ( \frac{c}{t} \Bigg ( \Vert y\Vert \Vert y - x' \Vert + \Vert y\Vert (t-t') + \ln (t) (t-t') \Bigg ) \Bigg ), \end{aligned}$$

for *t* so large that $t^{\sigma } \le \rho t$. Therefore,

$$\begin{aligned} \sum _{{x_1 + \cdots + x_l + x_{r+1}=x'}} \; \big ( q_{t_1}^{x_1} \big )^2 \cdots \big ( q_{t_l}^{x_l} \big )^2 \big ( q_{t_{r+1}}^{x_{r+1}} \big )^2&\lesssim t^{d/2} \ q_t^y \ q_{t'}^{x'} e^{\varphi (y - x')} \prod _{{j \in \{1, \ldots , l, r+1\}}} \biggl (c_1 t_j^{-d/2} \biggr ) \\&\lesssim t^{d/2} \ (q_t^y)^2 \ P (t) \prod _{{j \in \{1, \ldots , l, r+1\}}} \biggl (c_1 t_j^{-d/2} \biggr ), \end{aligned}$$

where $P (t) {:}=\exp \bigg ( \dfrac{c'}{t} \Big ( 2 \Vert y\Vert \Vert y - x' \Vert + \Vert y\Vert (t-t') + \ln (t) (t-t') \Big ) \bigg )$ for a constant $c' > 0$. In the second line of the estimate above, we also used that $\Vert \varphi \Vert \le \rho _2 \Vert y\Vert /t$.

Together with Lemma 14 from the appendix, we obtain the following estimate on $M^{t'}_{t,r,l} (x')$:

$$\begin{aligned} M^{t'}_{t,r,l} (x')&\lesssim t^{d/2} \ (q_t^y)^2 \ P (t) \qquad \sum _{{ \begin{array}{c} \\ t_1 + \cdots + t_l + t_{r+1} = t' \\ t_{r+1} \ge t_1, \ldots , t_l \ge t^\xi \end{array}}} \qquad \quad \; \prod _{j \in \{1, \ldots , l, r+1\}} \biggl (c_1 t_j^{-d/2} \biggr ) \\&\lesssim (q_t^y)^2 \ C^l \ t^{- \xi l (d-2) / 2} \left( \frac{t}{t'} \right) ^{d/2} P(t), \\&\le t^{- \xi /4} \ (q_t^y)^2 \ C^l \ t^{- \xi l (2d-5) / 4} \left( \frac{t}{t'} \right) ^{d/2} P(t), \end{aligned}$$

where $C > 0$ is a constant. It remains to bound $(t / t')^{d/2} P(t)$. We estimate the following expressions involved in $(t / t')^{d/2} P(t)$ like so:

$$\begin{aligned}{} & {} \left( \frac{t}{t'} \right) ^{d/2} \le \exp \left( \frac{d}{2} \ln (t) \frac{t - t'}{t-1} \right) , \qquad \quad t - t' = \sum _{j = l+1}^r t_j \le (r-l) t^\xi , \\{} & {} \quad \Vert y - x' \Vert \le \sum _{j = l+1}^r \Vert x_j \Vert \le \sum _{j = l+1}^r t_j \le (r-l) t^\xi , \end{aligned}$$

where $\sum _{j=l+1}^r \Vert x_j \Vert \le \sum _{j=l+1}^r t_j$ is valid under the assumption that $q_{t_1}^{x_1} \ldots q_{t_{r+1}}^{x_{r+1}} > 0$. Then, using $\Vert y\Vert \le t^{\sigma }$, we obtain

$$\begin{aligned} \left( \frac{t}{t'} \right) ^{d/2} P(t) \le \exp \big ( C' (r-l) t^{\sigma + \xi - 1} \big ) \end{aligned}$$

for some constant $C' > 0$. This completes the proof of Claim 4.1. $\square$

### Proof of the Central Lemma, Part 3: The Main Contribution

Let *t* be so large that $k(t) \ge 1$ and let $y \in \mathbb {Z}^d$ such that $\Vert y \Vert \le t^{\sigma }$ and $q^y_t > 0$. For $\textbf{i}\in I_1(t,r,m)$ and $\textbf{z}\in (\mathbb {Z}^d)^r$, define

29 $$\begin{aligned} q_{t,{\hat{m}}}^y(\textbf{i},\textbf{z}) := q_{i_1}^{z_1} \ldots \widehat{ q_{i_{m} - i_{m-1}}^{z_{m}-z_{m-1}} }\ldots q_{t-i_r}^{y-z_r}, \end{aligned}$$

where the factor with the hat is absent; in other words, we set the transition probability corresponding to the huge gap equal to 1.

Now decompose $B_3^{y,t}$ further, depending on the position of the huge gap 1) at the begining, 2) in the middle, or 3) at the end, as follows:

$$\begin{aligned} B_3^{y,t} = q_{t}^y \sum _{i=1}^3 \left( F_i^{y,t} + L_i^{y,t} \right) , \end{aligned}$$

where

30 $$\begin{aligned} F_{1}^{y,t} {:}=&\sum _{1 \le r \le k(t)} \sum _{\textbf{i}\in I_1(t, r,1), \textbf{z}} q_{t,{\hat{1}}}^y(\textbf{i}, \textbf{z}) \prod _{j=1}^r h(z_j, i_j), \nonumber \\ F_2^{y,t} {:}=&\sum _{2 \le r \le k(t)} \sum _{m=2}^r \; \sum _{\textbf{i}\in I_1(t,r,m), \textbf{z}} q_{t,{\hat{m}}}^y(\textbf{i}, \textbf{z}) \prod _{j=1}^r h(z_j, i_j), \nonumber \\ F_3^{y,t} {:}=&\sum _{1 \le r \le k(t)} \; \sum _{\textbf{i}\in I_1(t,r,r+1), \textbf{z}} q_{t,\widehat{r+1}}^y(\textbf{i}, \textbf{z}) \prod _{j=1}^r h(z_j, i_j); \end{aligned}$$

and the error terms are given by

$$\begin{aligned} L_1^{y,t} {:}=&\sum _{1 \le r \le k(t)} \; \sum _{\textbf{i}\in I_1(t,r,1), \textbf{z}} \frac{q_{i_1}^{z_1} - q_{t}^{y}}{q^{y}_{t}} q_{t,{\hat{1}}}^y(\textbf{i}, \textbf{z}) \prod _{j=1}^r h(z_j, i_j), \\ L_2^{y,t} {:}=&\sum _{2 \le r \le k(t)} \sum _{m=2}^r \; \sum _{\textbf{i}\in I_1(t,r,m), \textbf{z}} \frac{q_{i_m - i_{m-1}}^{z_m - z_{m-1}} - q_{t}^{y}}{q_{t}^{y}} q_{t,{\hat{m}}}^y(\textbf{i}, \textbf{z}) \prod _{j=1}^r h(z_j, i_j), \\ L_3^{y,t} {:}=&\sum _{1 \le r \le k(t)} \; \sum _{\textbf{i}\in I_1(t,r,r+1), \textbf{z}} \frac{q_{t-i_r}^{y-z_r}- q_{t}^{y}}{q^{y}_{t}} q_{t,\widehat{r+1}}^y(\textbf{i}, \textbf{z}) \prod _{j=1}^r h(z_j, i_j). \end{aligned}$$

Notice that $F_1^{y,t}, F_2^{y,t}, F_3^{y,t}$ are well-defined even if $q^y_t = 0$. We first show that the contribution from each error term is negligible.

#### Lemma 11

There exists $\theta > 0$ such that

$$\begin{aligned} \lim _{t \rightarrow \infty } t^{\theta } \sup _{y: \Vert y\Vert \le t^{\sigma }, q^y_t > 0} \biggl \langle \biggl |\sum _{i=1}^3 L_i^{y,t} \biggr |\biggr \rangle = 0. \end{aligned}$$

#### Proof

It is enough to show that there exists $\theta > 0$ such that for $i \in \{1,2,3\}$,

31 $$\begin{aligned} \lim _{t \rightarrow \infty } t^{\theta } \sup _{y: \Vert y\Vert \le t^{\sigma }, q^y_t > 0} \left\langle \left( L_i^{y,t} \right) ^2 \right\rangle = 0. \end{aligned}$$

For *t* so large that $k(t) \ge 1$ and for $y \in \mathbb {Z}^d$ such that $\Vert y \Vert \le t^{\sigma }$ and $q^y_t > 0$, one has

$$\begin{aligned} \left\langle \left( L_i^{y,t} \right) ^2 \right\rangle = \sum _{1 \le r \le k(t)} \lambda ^r a_i(r) \sum _{\begin{array}{c} \textbf{t}\in \Xi _r^i, \textbf{x} \end{array}} \left( q_{t_1}^{x_1} \right) ^2 \ldots \left( q_{t_r}^{x_r} \right) ^2 \frac{(q_{t-t_1 - \cdots - t_r}^{y-x_1 -\cdots -x_r} - q_t^y)^2}{(q_t^y)^2}, \end{aligned}$$

where $$a_i(r) {:}=1$$ if $$i=1,3$$, $$a_2(r) {:}=(r-1) \mathbbm {1}_{r \ge 2}$$,

$$\begin{aligned} \Xi _r^i {:}=&\left\{ \textbf{t}= (t_1,\ldots , t_r) \in \mathbb {N}_0^r ~:~ \begin{array}{c} \displaystyle t_1 \ge 0, t_2,\ldots , t_r> 0 \\ \displaystyle t_1 + \cdots + t_r \le r t^\xi \end{array}\right\} , \quad i=1,3, \\ \Xi _r^2 {:}=&\left\{ \textbf{t}= (t_1,\ldots , t_r) \in \mathbb {N}_0^r ~:~ \begin{array}{c} \displaystyle t_1, t_r \ge 0, t_2, \ldots , t_{r-1} > 0 \\ \displaystyle t_1 + \cdots + t_r \le r t^\xi \end{array} \right\} , \end{aligned}$$

and where the sum $\sum _{\textbf{x}}$ is taken over all $\textbf{x}= (x_1, \ldots , x_r) \in (\mathbb {Z}^d)^r$.

The convergence in (31) relies on $q_{t-t_1 -\cdots -t_r}^{y-x_1 -\cdots - x_r}$ being close to $q_t^y$ in the following sense: Let $\rho > 0$ be the constant from Lemma 9, and assume that *t* is so large that $t^{\sigma } \le \rho t$. Let $1 \le r \le k(t)$, $t_1, t_r \in \mathbb {N}_0$, $t_2, \ldots , t_{r-1} \in \mathbb {N}$ with $t_1 + \cdots + t_r \le r t^{\xi }$. Without loss of generality, let $x_1, \ldots , x_r \in \mathbb {Z}^d$ such that $q_{t_1}^{x_1} \ldots q_{t_r}^{x_r} > 0$, as otherwise the contribution to $\langle (L_i^{y,t})^2 \rangle$ is zero.

#### Claim 4.2

There exists a constant $c_3 > 0$ such that

$$\begin{aligned} \left( \frac{q_{t-t_1-\cdots -t_r}^{y-x_1-\cdots -x_r} - q_t^y}{q_t^y} \right) ^2 \le \left( 1 + O(t^{-\frac{2}{5}}) \right) \exp \left( c_3 r t^{\sigma +\xi -1} \right) - 1. \end{aligned}$$

Using this claim we can bound $\sup _{y: \Vert y\Vert \le t^{\sigma }, q^y_t > 0} \langle (L_i^{y,t})^2 \rangle$ by

32 $$\begin{aligned}&\sum _{r=1}^{\infty } \lambda ^r r \sum _{(t_1, \ldots , t_r) \in \mathbb {N}^{r}, \textbf{x}} \left( q_{t_1}^{x_1} \right) ^2 \ldots \left( q_{t_r}^{x_r} \right) ^2 \left( \left( 1 + O(t^{-\frac{2}{5}}) \right) \exp \left( c_3 r t^{\sigma +\xi -1} \right) - 1 \right) \nonumber \\&\quad \lesssim \sum _{r=1}^{\infty } \left( \alpha _d \lambda \right) ^r r \left( \left( 1 + O(t^{-\frac{2}{5}}) \right) \exp \left( c_3 r t^{\sigma +\xi -1} \right) - 1 \right) . \end{aligned}$$

Let $\theta \in (0, \min \{2/5, 1 - \sigma - \xi \})$. The definition of the Landau symbol $O(t^{-\frac{2}{5}})$ implies that there exist constants $C, T > 0$ such that for every $t > T$,

$$\begin{aligned}&t^{\theta } \left( \left( 1 + O(t^{-\frac{2}{5}}) \right) \exp \left( c_3 r t^{\sigma + \xi - 1} \right) - 1 \right) \\&\quad \le c_3 r t^{\sigma + \xi - 1 + \theta } \ \frac{\exp (c_3 r t^{\sigma + \xi - 1})-1}{c_3 r t^{\sigma + \xi - 1}} + C t^{-\frac{2}{5} + \theta } \exp \left( c_3 r t^{\sigma + \xi - 1} \right) \\&\quad \le \left( c_3 r + C \right) \exp \left( c_3 r t^{\sigma + \xi - 1} \right) , \end{aligned}$$

where, in the third line, we used that $(e^x - 1)/x \le e^x$ for every $x > 0$. Hence,

$$\begin{aligned}&t^{\theta } \sum _{r=1}^{\infty } \ \biggl |\left( \alpha _d \lambda \right) ^r r \left( \left( 1 + O(t^{-\frac{2}{5}}) \right) \exp \left( c_3 r t^{\sigma + \xi - 1} \right) - 1 \right) \biggr |\\&\quad \le \sum _{r=1}^{\infty } \left( \alpha _d \lambda \exp \left( c_3 t^{\sigma + \xi - 1} \right) \right) ^r \left( c_3 r^2 + C r \right) . \end{aligned}$$

For $\varphi \in (\alpha _d \lambda , 1)$ and *t* so large that $\alpha _d \lambda \exp (c_3 t^{\sigma + \xi -1}) \le \varphi$, the series on the right is dominated by the convergent series $\sum \varphi ^r (c_3 r^2 + Cr)$. Dominated convergence and (32) then imply (31) for $\theta \in (0, \max \{2/5, 1 - \sigma - \xi \})$.

To complete the proof of Lemma 11, it remains to prove Claim 4.2.

#### Proof of Claim 4.2

Let $x' {:}=x_1+\cdots + x_r$ and $t' {:}=t_1 + \cdots + t_r$. Observe that $q_{t - t'}^{y - x'} > 0$: Indeed, notice first that $t - t' \ge t - k(t) t^{\xi }$ since $t' \le r t^\xi$. As *k*(*t*) is of order $t^{\kappa _1}$ and $\kappa _1 + \xi < 1$, the term $t - t'$ is of order *t*. Moreover,

$$\begin{aligned} \Vert y - x' \Vert \le t^{\sigma } + \sum _{j=1}^r \Vert x_j\Vert \le t^{\sigma } + \sum _{j=1}^r t_j \le t^{\sigma } + k(t) t^{\xi }, \end{aligned}$$

which is of smaller order than $t - t'$. Finally, $t - t'$ and $\Vert y - x' \Vert _1$ have the same parity because $q^y_t > 0$ and $q_{t_1}^{x_1} \ldots q_{t_r}^{x_r} > 0$.

Now, we derive an upper bound on $|q_{t-t'}^{y-x'} - q_t^y |/{q_t^y}$. If $q_{t-t'}^{y-x'} \ge q_t^y$, then combining Lemma 9 with the estimate $\Vert x' \Vert \le t' \le r t^{\xi }$ gives

33 $$\begin{aligned} \frac{|q_{t-t'}^{y-x'} - q_t^y |}{q_t^y} \le&\left( 1+O(t^{-\frac{2}{5}}) \right) \exp \left( c \left( 2 t^{\sigma -1} r t^{\xi } + \frac{\ln (t)}{t} r t^{\xi } \right) \right) - 1 \nonumber \\ \le&\left( 1+O(t^{-\frac{2}{5}}) \right) \exp \left( c_1 r t^{\sigma +\xi -1} \right) - 1 \end{aligned}$$

for some constant $c_1 > 0$. If $q_t^y > q_{t-t'}^{y-x'}$, we argue as follows: Let $t \in \mathbb {N}$ be so large that $t^{\sigma } + k(t) t^{\xi } \le \rho (t-t').$ Then

$$\begin{aligned} \Vert y-x'\Vert \le t^{\sigma } + k(t) t^{\xi } \le \rho (t-t') \end{aligned}$$

and Lemma 9 with the estimate $k(t) t^{\xi } \lesssim t^{\kappa _1 + \xi } \le t^{\sigma }$ (coming from $\xi< \kappa _2 - \kappa _1 < \sigma - \kappa _1$) yields

34 $$\begin{aligned} \frac{|q_{t-t'}^{y-x'} - q_t^y |}{q_t^y}&\le \frac{q_t^y}{q_{t-t'}^{y-x'}} - 1 \nonumber \\&\le \left( 1 + O(t^{-\frac{2}{5}}) \right) \exp \left( c \left( \frac{t^{\sigma } + k(t) t^{\xi }}{t-t'} 2 r t^{\xi } + \ln (t-t') \frac{r t^{\xi }}{t-t'} \right) \right) -1 \nonumber \\&\le \left( 1 + O(t^{-\frac{2}{5}}) \right) \exp \left( c_2 r t^{\sigma +\xi -1} \right) -1. \end{aligned}$$

Using that $(a-1)^2 \le a^2 - 1$ for every $a \ge 1$, in either case (33) or (34), we have the following bound:

$$\begin{aligned} \left( \frac{q_{t-t'}^{y-x'} - q_t^y}{q_t^y} \right) ^2 \le \left( 1 + O\left( t^{-\frac{2}{5}}\right) \right) \exp \left( c_3 r t^{\sigma +\xi -1} \right) - 1, \end{aligned}$$

where $c_3 > 0$ is a constant. $\square$

In order to deal with the $F_i$’s defined in (30), the strategy is to first define suitable truncations of the partition functions. Fix $\xi _1, \xi _2$ satisfying

$$\begin{aligned} 0< \xi _1< \xi _2 < \xi , \end{aligned}$$

and notice that since $\xi + \sigma < 1$, we have $\xi _1 + \sigma < 1$. Now set

$$\begin{aligned} T_{0,0}^t := 1 + \sum _{1 \le r \le t^{\xi _1} +1} \sum _{\begin{array}{c} \textbf{i}\in I(t,r), \textbf{z}\\ i_r \le t^{\xi _2} \end{array}} q^y_{t, \widehat{r+1}}(\textbf{i}, \textbf{z}) \prod _{j=1}^r h(z_j, i_j) \end{aligned}$$

and

$$\begin{aligned} T_0^{y,t} := 1 + \sum _{1 \le r \le t^{\xi _1} + 1} \sum _{\begin{array}{c} \textbf{i}\in I(t,r), \textbf{z}\\ t - t^{\xi _2} \le i_1 \end{array}} q^y_{t, {\hat{1}}}(\textbf{i}, \textbf{z}) \prod _{j=1}^r h(z_j, i_j), \end{aligned}$$

where $q^y_{t, \widehat{r+1}}(\textbf{i}, \textbf{z})$ and $q^y_{t, {\hat{1}}}(\textbf{i}, \textbf{z})$ are defined according to (29), with $q^y_{t, \widehat{r+1}}(\textbf{i}, \textbf{z})$ not depending on *y*. Notice that $T_{0,0}^t$ and $T_0^{y,t}$ are truncations of the partition functions $Z_{0,0}^t$ and $Z_0^{y,t}$, respectively (see (4) and (3)). The convergence statement in (21) will follow from the lemmas below.

#### Lemma 12

There exists $\theta > 0$ such that

35 $$\begin{aligned} \lim _{t \rightarrow \infty } t^{\theta } \sup _{\Vert y\Vert \le t^{\sigma }} \left\langle \left|F_2^{y,t} - (T_{0,0}^t - 1) (T^{y,t}_0 - 1) \right|\right\rangle =&0, \end{aligned}$$

36 $$\begin{aligned} \lim _{t \rightarrow \infty } t^{\theta } \sup _{\Vert y\Vert \le t^{\sigma }} \left\langle \left|F_1^{y,t} - (T^{y,t}_0 - 1) \right|\right\rangle =&0, \end{aligned}$$

37 $$\begin{aligned} \lim _{t \rightarrow \infty } t^{\theta } \sup _{\Vert y\Vert \le t^{\sigma }} \left\langle \left|F_3^{y,t} - (T_{0,0}^t - 1) \right|\right\rangle =&0. \end{aligned}$$

#### Lemma 13

There exists $\theta > 0$ such that

38 $$\begin{aligned} \lim _{t \rightarrow \infty } t^{\theta } \sup _{\Vert y\Vert \le t^{\sigma }} \left\langle \left|T^{y,t}_0 T_{0,0}^t - Z_{0,0}^{\infty } Z_{-\infty }^{y,t} \right|\right\rangle =&0. \end{aligned}$$

The convergence statement in (35) is shown in Sect. 5.1. We show the convergence statements in (36) and (37) in Sect. 5.2, and the one in (38) in Sect. 5.3.

## Main Contribution: Proofs of Lemmas 12 and 13

### Proof of Lemma 12, (35): Convergence for One Huge Gap in the Middle

One has

39 $$\begin{aligned}&(T_{0,0}^t - 1) (T^{y,t}_0 - 1) \nonumber \\&\quad = \sum _{1 \le r \le t^{\xi _1}+1} \sum _{1 \le s \le t^{\xi _1}+1} \sum _{\begin{array}{c} 0 \le i_1< \cdots< i_r \le t^{\xi _2}, \\ z_1, \ldots , z_r \in \mathbb {Z}^d \end{array}} \sum _{\begin{array}{c} t - t^{\xi _2} \le l_1< \cdots < l_s \le t, \\ c_1, \ldots , c_s \in \mathbb {Z}^d \end{array}} \nonumber \\&\quad q_{i_1}^{z_1} \ldots q_{i_r - i_{r-1}}^{z_r - z_{r-1}} q_{l_2 - l_1}^{c_2 - c_1} \ldots q_{t - l_s}^{y - c_s} \prod _{j=1}^r h(z_j, i_j) \prod _{k=1}^s h(c_k, l_k). \end{aligned}$$

Define the set

$$\begin{aligned} V(t,r,m) {:}=\left\{ \textbf{i}= (i_1, \ldots , i_r) \in I_1(t,r,m) ~:~ \begin{array}{c} \displaystyle 0 \le i_1< \cdots< i_{m-1} \le t^{\xi _2} \\ \displaystyle \quad t - t^{\xi _2} \le i_m< \cdots < i_r \le t \end{array} \right\} \end{aligned}$$

and its complement in $I_1(t,r,m)$

$$\begin{aligned} W(t,r,m) {:}=\left\{ \textbf{i}= (i_1, \ldots , i_r) \in I_1(t,r,m) ~:~ \begin{array}{c} \displaystyle i_{m-1} > t^{\xi _2} \quad \text {or} \quad i_m < t - t^{\xi _2} \end{array} \right\} . \end{aligned}$$

Recall the notation $q^{y}_{t,{{\hat{m}}}}(\textbf{i},\textbf{z})$ from (29). Making the change of summation indices $r{:}=r+s$ and $m{:}=r+1$ in (39), one has

40 $$\begin{aligned}&(T_{0,0}^t - 1) (T^{y,t}_0 - 1) \nonumber \\&\quad =\hspace{-3mm} \sum _{2 \le r \le t^{\xi _1}+ 2} \ \sum _{m=2}^r \ \sum _{\textbf{i}\in V(t,r,m), \textbf{z}} q_{t,{{\hat{m}}}}^y(\textbf{i},\textbf{z}) \prod _{j=1}^r h(z_j, i_j) \nonumber \\&\qquad + \sum _{\begin{array}{c} t^{\xi _1}+ 2 < r, \\ r \le 2 t^{\xi _1}+ 2 \end{array}} \ \sum _{\begin{array}{c} r - t^{\xi _1} \le m, \\ m \le t^{\xi _1} + 2 \end{array}} \ \sum _{\textbf{i}\in V(t,r,m), \textbf{z}} q_{t,{{\hat{m}}}}^y(\textbf{i},\textbf{z}) \prod _{j=1}^r h(z_j, i_j). \end{aligned}$$

The identity in (40) allows us to rewrite $$F_2^{y,t} - (T_{0,0}^t - 1) (T_0^{y,t} - 1)$$ as $$ f^{y,t}_{2;1} + f^{y,t}_{2;2} + f^{y,t}_{2;3}, $$ where

$$\begin{aligned} f^{y,t}_{2;1} {:}=&\sum _{r \in R^1} \ \sum _{m = 2}^r \ \sum _{\textbf{i}\in W(t,r,m), \textbf{z}} q^{y}_{t,{{\hat{m}}}}(\textbf{i},\textbf{z}) \prod _{j=1}^r h(z_j, i_j), \\ f^{y,t}_{2;2} {:}=&\sum _{r \in R^2} \biggl ( \sum _{m=2}^r \sum _{\textbf{i}\in I_1(t,r,m), \textbf{z}} q^y_{t, {\hat{m}}}(\textbf{i}, \textbf{z}) \prod _{j=1}^r h(z_j, i_j) \\&- \sum _{\begin{array}{c} r - t^{\xi _1} \le m, \\ m \le t^{\xi _1}+ 2 \end{array}} \ \sum _{\textbf{i}\in V(t,r,m), \textbf{z}} q_{t,{{\hat{m}}}}^y(\textbf{i},\textbf{z}) \prod _{j=1}^r h(z_j, i_j) \biggr ), \\ f^{y,t}_{2;3} {:}=&\sum _{r \in R^3} \ \sum _{m=2}^r \ \sum _{\textbf{i}\in I_1(t,r,m), \textbf{z}} q^{y}_{t,{{\hat{m}}}}(\textbf{i},\textbf{z}) \prod _{j=1}^r h(z_j, i_j), \end{aligned}$$

$$R^1 {:}=\{r \in \mathbb {N}: \ 2 \le r \le t^{\xi _1} + 2\}$$, $$R^2 {:}=\{r \in \mathbb {N}: \ t^{\xi _1} + 2 < r \le 2 t^{\xi _1} + 2\}$$, and $$R^3 {:}=\{r \in \mathbb {N}: \ 2 t^{\xi _1} + 2 < r \le k(t)\}$$.

In order to prove (35), it is then enough to show existence of $\theta > 0$ such that for $i=1,2,3$,

41 $$\begin{aligned} \lim _{t \rightarrow \infty } t^{\theta } \sup _{\Vert y\Vert \le t^{\sigma }} \left\langle \left( f_{2;i}^{y,t} \right) ^2 \right\rangle = 0. \end{aligned}$$

For $i = 1, 3$, one has

42 $$\begin{aligned} \langle (f_{2;i}^{y,t})^2 \rangle = \sum _{r \in R^i} \lambda ^r \sum _{m=2}^r \ \sum _{\textbf{i}\in H^i(t,r,m), \textbf{z}} q^{y}_{t,{{\hat{m}}}}(\textbf{i},\textbf{z})^2, \end{aligned}$$

where $H^1(t,r,m) {:}=W(t,r,m)$ and $H^3(t,r,m) {:}=I_1(t,r,m)$. Notice furthermore that $\langle (f_{2;2}^{y,t})^2 \rangle$ is bounded by (42) with $i=2$ and $H^2(t,r,m) {:}=I_1(t,r,m)$. Now, we take up cases $i=1,2,3$ separately.

**Case** $i = 1$. Since for $\textbf{i}\in W(t,r,m)$,

$$\begin{aligned} i_1 + (i_2 - i_1)&+ \cdots + (i_{m-1} - i_{m-2}) + (i_{m+1} - i_m) + \cdots + (t - i_r) \\ =&i_{m-1} - i_m + t \ge \max \{i_{m-1}; t-i_m\} > t^{\xi _2}, \end{aligned}$$

the expression in (42) is dominated by

$$\begin{aligned} \sum _{r \in R^1} r \lambda ^r \sum _{\begin{array}{c} t_1, \ldots , t_r \in \mathbb {N}, \textbf{x}\\ t_1+ \cdots + t_r> t^{\xi _2} \end{array}} \left( q_{t_1}^{x_1} \right) ^2 \ldots \left( q_{t_r}^{x_r} \right) ^2 \lesssim \sum _{r=1}^{\infty } r^2 (\alpha _d \lambda )^{r} \sum _{j > \frac{t^{\xi _2}}{t^{\xi _1}+2}} \frac{1}{j^{\frac{d}{2}}} \lesssim t^{(\xi _2 - \xi _1) \left( 1-\frac{d}{2}\right) }. \end{aligned}$$

This implies (41) for $\theta < (\xi _2-\xi _1) (\tfrac{d}{2}-1)$.

**Case** $i=2$. The expression in (42) is dominated by

$$\begin{aligned} \sum _{r \in R^2} r \lambda ^r \sum _{t_1, \ldots , t_{r} \in \mathbb {N}, \textbf{x}} \left( q_{t_1}^{x_1} \right) ^2 \ldots \left( q_{t_{r}}^{x_{r}} \right) ^2 \le \sum _{r > t^{\xi _1}} r (\alpha _d \lambda )^r. \end{aligned}$$

From this estimate we deduce (41) for every $\theta > 0$.

**Case** $i = 3$. The expression in (42) is dominated by

$$\begin{aligned} \sum _{r \in R^3} r (\alpha _d \lambda )^r, \end{aligned}$$

which converges to 0 as $t\rightarrow \infty$ faster than any polynomial by the same argument as in the case $i=2$.

### Proof of Lemma 12, (36) and (37): Convergence for One Huge Gap at the Start or the End

We only show the convergence statement in (37) as the proof of (36) is analogous. Write

$$\begin{aligned} F_3^{y,t} - T_{0,0}^t + 1 = f_{3;1}^t + f_{3;2}^t, \end{aligned}$$

where for $$i= 1,2$$,

$$\begin{aligned} f_{3;i}^t {:}=&\sum _{r \in R^i} \sum _{\begin{array}{c} \textbf{i}\in H^i(t,r), \textbf{z} \end{array}} q^y_{t, \widehat{r+1}}(\textbf{i},\textbf{z}) \prod _{j=1}^r h(z_j, i_j) \end{aligned}$$

and $$R^1 {:}=\{r \in \mathbb {N}: \ 1 \le r \le t^{\xi _1}+1\}$$, $$R^2 {:}=\{r \in \mathbb {N}: \ t^{\xi _1}+1 < r \le k(t) \}$$,

$$H^1(t,r) {:}=\left\{ \textbf{i}= (i_1, \ldots , i_r) ~:~ \begin{array}{c} \displaystyle 0 \le i_1< \cdots < i_{r} \le r t^{\xi } \\ \displaystyle i_r > t^{\xi _2} \end{array} \right\} , $$ $$ H_r^2 {:}=I_1(t,r,r+1). $$

For $i = 1,2$, one has

$$\begin{aligned} \left\langle \left( f_{3;i}^t \right) ^2 \right\rangle = \sum _{r \in R^i} \lambda ^r \sum _{\textbf{i}\in H^i(t,r), \textbf{z}} q^y_{t, \widehat{r+1}}(\textbf{i}, \textbf{z})^2. \end{aligned}$$

Convergence in the cases $i=1$ and $i=2$ works then as in the proof of (35).

### Proof of Lemma 13: Convergence to Limiting Partition Functions

Let us first show that the truncated partition function $T_{0,0}^t$ converges to the limiting partition function $Z_{0,0}^\infty$ in the $L^2$ sense and obtain a rate of convergence. We will prove that there exists $\theta > 0$ such that

43 $$\begin{aligned} \lim _{t \rightarrow \infty } t^{\theta } \left\langle \left( T_{0,0}^t - Z_{0,0}^t \right) ^2 \right\rangle = 0. \end{aligned}$$

One has

$$\begin{aligned} Z_{0,0}^t - T_{0,0}^t = N_1^t + N_2^t, \end{aligned}$$

where

$$\begin{aligned} N_1^t {:}=&\sum _{1 \le r \le t^{\xi _1} + 1} \sum _{\begin{array}{c} \textbf{i}\in I(t,r), \textbf{z}\\ i_r > t^{\xi _2} \end{array}} q^y_{t, \widehat{r+1}}(\textbf{i},\textbf{z}) \prod _{j=1}^r h(z_j, i_j), \\ N_2^t {:}=&\sum _{t^{\xi _1} + 1 < r \le t+1} \sum _{\textbf{i}\in I(t,r), \textbf{z}} q^y_{t, \widehat{r+1}}(\textbf{i}, \textbf{z}) \prod _{j=1}^r h(z_j, i_j). \end{aligned}$$

It is then enough to show existence of $\theta > 0$ such that

44 $$\begin{aligned} \lim _{t \rightarrow \infty } t^{\theta } \left\langle \left( N_i^t \right) ^2 \right\rangle = 0, \quad i \in \{1,2\}. \end{aligned}$$

We have

$$\begin{aligned} \left\langle \left( N_2^t \right) ^2 \right\rangle = \sum _{t^{\xi _1} + 1 < r \le t+1} \lambda ^r \sum _{\textbf{i}\in I(t,r), \textbf{z}} q^y_{t, \widehat{r+1}}(\textbf{i}, \textbf{z})^2 \lesssim \sum _{r > t^{\xi _1} + 1} (\alpha _d \lambda )^r, \end{aligned}$$

so (44) holds for $i=2$ and for every $\theta > 0$. Moreover,

$$\begin{aligned} \left\langle \left( N_1^t \right) ^2 \right\rangle =&\sum _{1 \le r \le t^{\xi _1} +1} \lambda ^r \sum _{\begin{array}{c} \textbf{i}\in I(t,r), \textbf{x}\\ i_r> t^{\xi _2} \end{array}} q^y_{t, \widehat{r+1}}(\textbf{i},\textbf{z})^2, \\ \lesssim&\sum _{1 \le r \le t^{\xi _1} +1} \lambda ^r \sum _{\begin{array}{c} t_1, \ldots , t_r \in \mathbb {N}, \textbf{x}\\ t_1 + \cdots + t_r > t^{\xi _2} \end{array}} \left( q_{t_1}^{x_1} \right) ^2 \ldots \left( q_{t_r}^{x_r} \right) ^2, \end{aligned}$$

so (44) holds for $i=1$ and $\theta \in (0, (\xi _2 - \xi _1) (\tfrac{d}{2}-1))$. This implies (43). Combining (43) with Theorem 2, one obtains in particular that there exists $\theta > 0$ such that

45 $$\begin{aligned} \lim _{t \rightarrow \infty } t^{\theta } \left\langle \left( T_{0,0}^t - Z_{0,0}^{\infty } \right) ^2 \right\rangle = 0. \end{aligned}$$

To complete the proof of Lemma 13, notice that

$$\begin{aligned} \left\langle \left|T_{0,0}^t T^{y,t}_0 - Z_{0,0}^{\infty } Z_{-\infty }^{y,t} \right|\right\rangle \le \left\langle \left|T^{y,t}_0 \left( T_{0,0}^t - Z_{0,0}^{\infty } \right) \right|\right\rangle + \left\langle \left|Z_{0,0}^{\infty } \left( T^{y,t}_0 - Z_{-\infty }^{y,t} \right) \right|\right\rangle . \end{aligned}$$

Therefore, we obtain the desired result by applying the Cauchy-Schwarz Inequality to the two summands on the right-hand side, and using (45) together with

$$\begin{aligned} \lim _{t \rightarrow \infty } \left\langle \left( T_{0,0}^t \right) ^2 \right\rangle = \left\langle \left( Z_{0,0}^{\infty } \right) ^2 \right\rangle < \infty . \end{aligned}$$

## Data Availability

Data sharing does not apply to this article as no datasets were generated or analyzed during the current study.

## Appendix A Proofs of Estimates for Transition Probabilities

### A.1 Proof of Lemma 6

For $t \in \mathbb {N}_0$, set $\gamma _t^* {:}=\gamma _{2t}$. Then $\gamma ^*$ is a random walk on the lattice $(\mathbb {Z}^d)_{\text {ev}}$ consisting of those points in $\mathbb {Z}^d$ whose coordinate sum is even. If $\{ e_j \}_{1 \le j \le d}$ is the standard basis for ${\mathbb {R}}^d$, then $\{e_1 + e_j: 1 \le j \le d\}$ is a basis for $(\mathbb {Z}^d)_{\text {ev}}$. Let $L : {\mathbb {R}}^d \rightarrow {\mathbb {R}}^d$ be the linear transformation mapping $e_1 + e_j$ to $e_j$ for $1 \le j \le d$, and define ${\tilde{\gamma }}_t {:}=L \gamma _t^*$. Then, ${{\tilde{\gamma }}}$ is an aperiodic, irreducible, symmetric random walk on $\mathbb {Z}^d$ with bounded increments, so it satisfies the conditions of Theorem 2.3.11 in [11]. Thus, there exists $\rho > 0$ such that for every $i \in \mathbb {N}$ and $z \in \mathbb {Z}^d$ satisfying $\Vert z\Vert < \rho i$, we have

$$\begin{aligned} {{\tilde{q}}}_i^z {:}=\textbf{P}({{\tilde{\gamma }}}_i = z) = 2 \left( \frac{d}{4 \pi i} \right) ^{\frac{d}{2}} \exp \left( - \frac{d}{4i} \Vert L^{-1} z \Vert ^2 \right) \exp \left( O \left( \frac{1}{i} + \frac{\Vert z\Vert ^4}{i^3} \right) \right) . \end{aligned}$$

Now, fix $\sigma \in (\tfrac{3}{4}, 1)$, ${\tilde{\sigma }} \in (\sigma ,1)$, and let $T \in \mathbb {N}$ be so large that $1 + t^{\sigma } < (t-1)^{{{\tilde{\sigma }}}}$ and $t^{{{\tilde{\sigma }}}} < \tfrac{\rho }{2 {\left| \hspace{-1.0625pt}\left| \hspace{-1.0625pt}\left| L \right| \hspace{-1.0625pt}\right| \hspace{-1.0625pt}\right| } } t$ for every $t \ge T$, where ${\left| \hspace{-1.0625pt}\left| \hspace{-1.0625pt}\left| L \right| \hspace{-1.0625pt}\right| \hspace{-1.0625pt}\right| }$ is the operator norm of *L*. We distinguish between two cases: *t* is either even or odd.

Even case. If $t=2m$ for some $m \in \mathbb {N}$, then we can prove a slightly stronger statement:

#### Claim A.1

There exist constants $c_1, c_2 > 0$, independent of $\sigma$ and ${\tilde{\sigma }}$, such that (14) holds for every even $t \ge T$ and $y \in \mathbb {Z}^d$ with $q^y_{t} > 0$ and $\Vert y\Vert \le {t}^{{{\tilde{\sigma }}}}$.

The difference to the conclusion of Lemma 6 is that the estimate holds for $\Vert y \Vert \le t^{{{\tilde{\sigma }}}}$ and not just for $\Vert y\Vert \le t^{\sigma }$. To prove this claim, fix $t=2m \ge T$ and $y \in \mathbb {Z}^d$ such that $q^y_{2m} > 0$ and $\Vert y\Vert \le (2m)^{{{\tilde{\sigma }}}}$. Then $q^y_{2m} = {{\tilde{q}}}^{Ly}_m.$ Since $\Vert Ly \Vert \le {\left| \hspace{-1.0625pt}\left| \hspace{-1.0625pt}\left| L \right| \hspace{-1.0625pt}\right| \hspace{-1.0625pt}\right| } \Vert y\Vert \le {\left| \hspace{-1.0625pt}\left| \hspace{-1.0625pt}\left| L \right| \hspace{-1.0625pt}\right| \hspace{-1.0625pt}\right| } t^{{{\tilde{\sigma }}}} < \rho m,$ one has

$$\begin{aligned} {\tilde{q}}^{Ly}_m&= 2 \left( \tfrac{d}{2 \pi t} \right) ^{\frac{d}{2}} \exp \big ( -\tfrac{d}{2t} \Vert y\Vert ^2 \big ) \exp \left( O \left( \frac{1}{m} + \frac{\Vert Ly\Vert ^4}{m^3} \right) \right) \\&\ge c_1 \left( \tfrac{d}{2 \pi t} \right) ^{\frac{d}{2}} \exp \big ( -\tfrac{d}{2t} \Vert y\Vert ^2 \big ) \exp \big (-c_2 t^{4 {{\tilde{\sigma }}} -3} \big ) \end{aligned}$$

for some constants $c_1, c_2 > 0$.

Odd case. Now, suppose $t = 2m+1 \ge T$ for some $m \in \mathbb {N}$. Fix $y \in \mathbb {Z}^d$ such that $q^y_{2m+1} > 0$ and $\Vert y\Vert \le (2m+1)^{\sigma }$. Let *E* be the set of standard unit vectors in $\mathbb {R}^d$ and their additive inverses. Then

$$\begin{aligned} q^y_{2m+1} = \sum _{z \in \mathbb {Z}^d} q^{y-z}_{2m} q^z_1 = \frac{1}{2d} \sum _{z \in E} q^{y-z}_{2m}. \end{aligned}$$

Since $\Vert y-z\Vert< 1 + t^{\sigma } < (t-1)^{{{\tilde{\sigma }}}} = (2m)^{{{\tilde{\sigma }}}}$ and $q^{y-z}_{2m} > 0$ for every $z \in E$, then using Claim A.1, we can bound $q^y_{2m+1}$ from below as follows: There exist $c'_1, c'_2 > 0$ such that

A1 $$\begin{aligned} q^{y}_{2m+1}&\ge \frac{c'_1}{2d} \left( \tfrac{d}{4 \pi m} \right) ^{\frac{d}{2}} \exp \Big (-c'_2 (2m)^{4 {{\tilde{\sigma }}} -3} \Big ) \sum _{z \in E} \exp \big ( -\tfrac{d}{4m} \Vert y - z\Vert ^2 \big ) \nonumber \\&\ge \frac{c'_1}{2d} \left( \tfrac{d}{2 \pi t} \right) ^{\frac{d}{2}} \exp \Big (-c'_2 t^{4 {{\tilde{\sigma }}} -3} \Big ) \exp \big ( -\tfrac{d}{4m} \Vert y - e_1\Vert ^2 \big ). \end{aligned}$$

In addition to $t \ge T$, assume that *t* is so large that

$$\begin{aligned} \exp \left( -\frac{d}{2} \left( \frac{t^{2 \sigma }}{t(t-1)} + \frac{1+ 2 t^{\sigma }}{t-1} \right) \right) > \frac{1}{2}. \end{aligned}$$

Since $\Vert y - e_1 \Vert ^2 = \Vert y\Vert ^2 + 1 - 2 y \cdot e_1 \le \Vert y\Vert ^2 + 1 + 2 \Vert y\Vert ,$ it follows that

$$\begin{aligned} \exp \big ( -\tfrac{d}{4m} \Vert y - e_1\Vert ^2 \big ) \ge&\exp \Big (- \tfrac{d}{4m} \big (\Vert y\Vert ^2 + 1 + 2 \Vert y\Vert \big ) \Big ) \\ \ge&\exp \Big ( - \tfrac{d}{2t} \Vert y \Vert ^2 \Big ) \exp \Big (-\tfrac{d}{2} \Big (\tfrac{t^{2 \sigma }}{t(t-1)} + \tfrac{1 + 2 t^{\sigma }}{t-1} \Big ) \Big ) \\ >&\tfrac{1}{2} \exp \Big ( - \tfrac{d}{2t} \Vert y \Vert ^2 \Big ). \end{aligned}$$

Plugging this into the right-hand side of (A1), we obtain the desired estimate.

### A.2 Proof of Lemma 7

Recall from Sect. 3 that $\theta ^0 = (0,\ldots ,0)$ and $\theta ^1 = (\pi ,\ldots ,\pi )$. For $\varepsilon > 0$ and $j\in \{0,1\}$, let ${\mathcal {D}}_j^\varepsilon {:}=\{ \theta \in \mathbb {R}^d ~:~ \Vert \theta - \theta ^j \Vert < \varepsilon \}$. Let $\varphi$ be a linear functional on $\mathbb {R}^d$ such that $|\varphi (x) |\le \Vert x \Vert , x \in \mathbb {R}^d$, and let $\Phi$ be the corresponding function defined in (15).

#### Claim A.2

There exist $\varepsilon , \delta > 0$ such that, for $j \in \{0,1\}$,

$$\begin{aligned} \left|\frac{ \Phi (\theta ) }{\Phi ( \theta ^j ) } \right|\le e^{- \delta \Vert \theta - \theta ^j \Vert ^2} \qquad \,\quad {for every \theta \in \mathcal {C}\setminus {\mathcal {D}}_{1-j}^\varepsilon .} \end{aligned}$$

#### Proof of Claim A.2

For $j \in \{0,1\}$, define scaled versions of the gradient vector and the Hessian matrix of $\Phi$ at $\theta ^j$:

$$\begin{aligned} G_j {:}=-i \frac{ \nabla \Phi (\theta ^j) }{\Phi (\theta ^j)} \qquad \text {and}\qquad H_j {:}=- \frac{1}{2} \frac{\nabla ^2 \Phi (\theta ^j)}{\Phi (\theta ^j)}. \end{aligned}$$

A simple computation shows that the matrix $H_j$ is diagonal, and that for every $l \in \{ 1, \ldots , d \}$, the *l*-th component of $G_j$ and the (*l*, *l*)-entry of $H_j$ are, respectively,

A2 $$\begin{aligned} G_j^l = \frac{\sinh (\varphi _l)}{d \Phi (0)} \qquad \text {and}\qquad H_j^l = \frac{\cosh (\varphi _l)}{2d \Phi (0)}. \end{aligned}$$

If we Taylor expand $\Phi$ around $\theta ^j$, we obtain

$$\begin{aligned} \left|\frac{\Phi (\theta )}{\Phi (\theta ^j)} \right|&= \Big |1 + i G_j \cdot (\theta - \theta ^j) - (\theta - \theta ^j) \cdot H_j (\theta - \theta ^j) + O \big ( \Vert \theta - \theta ^j \Vert ^3 \big ) \Big |\\&= \Big ( 1 - 2 (\theta - \theta ^j) \cdot H_j (\theta - \theta ^j) + (G_j \cdot (\theta - \theta ^j))^2 + O \big ( \Vert \theta - \theta ^j \Vert ^3 \big ) \Big )^{1/2}. \end{aligned}$$

Here and in the sequel, $g(\theta ) = O(f(\theta ))$ means there exists a constant $c > 0$, independent of $\varphi$, such that $|g(\theta ) |\le c f(\theta )$. In the Taylor expansion above, the constant *c* corresponding to the error term $O\big ( \Vert \theta - \theta ^j \Vert ^3 \big )$ may be chosen independently of $\varphi$ because of the assumption that $\Vert \varphi \Vert \le 1$. Notice from (A2) that $G_0 = G_1$ and $H_0 = H_1$, so in order to prove Claim A.2, it is enough to consider the case $j = 0$, where $\theta ^j = (0, \ldots , 0)$. If we write $\theta = (\theta _1, \ldots , \theta _d)$, then, using Jensen’s Inequality for sums,

$$\begin{aligned} \big (G_0 \cdot \theta \big )^2 \le \frac{1}{d \Phi (0)} \sum _{l=1}^d \sinh ( |\varphi _l |) \ \theta _l^2. \end{aligned}$$

Using the expression for $H_0$ in (A2) as well as $\Vert \varphi \Vert \le 1$, we obtain

$$\begin{aligned} 2 \theta \cdot H_0 \theta - \big ( G_0 \cdot \theta \big )^2 \ge \frac{1}{d \Phi (0)} \sum _{l=1}^d e^{- |\varphi _l |} \theta _l^2 \ge \frac{1}{de \Phi (0)} \Vert \theta \Vert ^2. \end{aligned}$$

Thus, there exist $\varepsilon > 0$ and a constant $c > 0$ such that for every $\theta$ with $\Vert \theta \Vert \le \varepsilon$,

$$\begin{aligned} \left|\frac{\Phi (\theta )}{\Phi (0)} \right|\le \left( 1 - \frac{1}{de \Phi (0)} \Vert \theta \Vert ^2 + O \big ( \Vert \theta \Vert ^3 \big ) \right) ^{1/2} \le \big ( 1 - c \Vert \theta \Vert ^2 \big )^{1/2}. \end{aligned}$$

Since the map $\theta \mapsto \big |\Phi (\theta ) / \Phi (0) \big |$ is continuous and strictly less than 1 for every $\theta \in \mathcal {C}$ except $\theta ^0, \theta ^1$, it follows that

$$\begin{aligned} {\mathcalligra{s}}(\varphi ) {:}=\sup \left\{ \big |\Phi (\theta ) / \Phi (0) \big |: \ \theta \in \mathcal {C}; \ \Vert \theta \Vert , \Vert \theta - \theta ^1\Vert \ge \varepsilon \right\} < 1. \end{aligned}$$

In fact, one even has $\sup _{\Vert \varphi \Vert \le 1} {\mathcalligra{s}}(\varphi ) < 1$. Hence, if we choose ${\tilde{c}} \in (0, c)$ so small that $\big ( 1 - {\tilde{c}} \Vert \theta \Vert ^2 \big ) \ge (\sup _{\Vert \varphi \Vert \le 1} {\mathcalligra{s}}(\varphi ))^2$ for every $\theta \in \mathcal {C}$, then Claim A.2 follows with $\delta {:}={\tilde{c}} / 2$.

For $t \in \mathbb {N}$, let $\widehat{\Phi ^t}$ be the Fourier transform of $\Phi ^t$; i.e.,

$$\begin{aligned} \widehat{\Phi ^t}(z) {:}=\frac{1}{(2 \pi )^d} \int _{\mathcal {C}} \Phi (\theta )^t e^{-i \theta \cdot z} \, d \theta , \quad z \in \mathbb {Z}^d. \end{aligned}$$

Since $\Phi (\theta )^t = \textbf{E}\left[ e^{i \theta \cdot \gamma _t} e^{\varphi (\gamma _t)} \right] ,$ one has

A3 $$\begin{aligned} \widehat{\Phi ^t}(z) = \sum _{y \in \mathbb {Z}^d} \textbf{P}(\gamma _t = y) e^{\varphi (y)} \frac{1}{(2 \pi )^d} \int _{\mathcal {C}} e^{i \theta \cdot (y-z)} \, d \theta = q_t^z e^{\varphi (z)}. \end{aligned}$$

Now, we estimate with the help of (17) and Claim A.2:

$$\begin{aligned} q_t^z e^{\varphi (z)}&\le \frac{1}{(2\pi )^d} \int _\mathcal {C}\big |\Phi (\theta ) \big |^t \, d\theta = \frac{1}{(2\pi )^d} \int _\mathcal {C}\left|\frac{\Phi (\theta )}{\Phi (0)} \right|^t \, d\theta \ \sum _{y \in \mathbb {Z}^d} q_t^y e^{\varphi (y)} \\&\le \frac{1}{(2 \pi )^d} \left( \int _{\mathcal {C}\setminus {\mathcal {D}}_1^\varepsilon } e^{- \delta t \Vert \theta \Vert ^2} \, d\theta + \int _{\mathcal {C}\setminus {\mathcal {D}}_0^\varepsilon } e^{- \delta t \Vert \theta - \theta ^1 \Vert ^2} \, d\theta \right) \sum _{y \in \mathbb {Z}^d} q_t^y e^{\varphi (y)} \\&\le \frac{2}{(2\pi )^d} \int _{\mathbb {R}^d} e^{- \delta t \Vert \theta \Vert ^2} \, d\theta \ \sum _{y \in \mathbb {Z}^d} q_t^y e^{\varphi (y)}. \end{aligned}$$

Finally, for some constant $C > 0$,

$$\begin{aligned} \int _{\mathbb {R}^d} e^{-\delta t \Vert \theta \Vert ^2} \, d\theta \le C \int _0^{\infty } r^{d-1} e^{-\delta t r^2} \, dr = C t^{-\frac{d}{2}} \int _0^{\infty } \rho ^{d-1} e^{-\delta \rho ^2} \, d \rho . \end{aligned}$$

### A.3 Proof of Lemma 8

For $j \in \{0,1\}$, let ${\mathcal {B}}_j {:}=\{ \theta \in \mathcal {C}~:~ \Vert \theta - \theta ^j \Vert \le t^{-2/5} \}$. Recall from (A3) that for every $z \in \mathbb {Z}^d$, $t \in \mathbb {N}$, and for every linear functional $\varphi$ on $\mathbb {R}^d$ satisfying $\Vert \varphi \Vert \le 1$, one has

$$\begin{aligned} q_t^z e^{\varphi (z)} = \frac{1}{(2 \pi )^d} \int _{\mathcal {C}} \Phi (\theta )^t e^{-i \theta \cdot z} \, d \theta = I_0 + I_1 + I, \end{aligned}$$

where

$$\begin{aligned} I_j {:}=\frac{1}{(2 \pi )^d} \int _{{\mathcal {B}}_j} \Phi (\theta )^t e^{-i \theta \cdot z} \, d \theta \quad \text {and}\quad I {:}=\frac{1}{(2 \pi )^d} \int _{\mathcal {C}\setminus ({\mathcal {B}}_0 \cup {\mathcal {B}}_1) } \Phi (\theta )^t e^{-i \theta \cdot z} \, d \theta . \end{aligned}$$

Then we find

A4 $$\begin{aligned} \frac{1}{(2 \pi )^d} \int _{\mathcal {C}} \big |\Phi (\theta ) \big |^t \, d \theta - q^z_t e^{\varphi (z)}&\le \sum _{j \in \{0, 1\}} \left( \frac{1}{(2 \pi )^d} \int _{{\mathcal {B}}_j} \big |\Phi (\theta ) \big |^t \, d \theta - \text {Re}(I_j) \right) \nonumber \\&\quad + \frac{2}{(2\pi )^d} \int _{\mathcal {C}\setminus ({\mathcal {B}}_0 \cup {\mathcal {B}}_1)} \big |\Phi (\theta ) \big |^t \, d \theta . \end{aligned}$$

We now estimate the expression on the right-hand side. First, we show that the linear functional $\varphi$ can be chosen in such a way that for $j \in \{0,1\}$,

A5 $$\begin{aligned} \frac{1}{(2 \pi )^d} \int _{\mathcal {B}_j} \big |\Phi (\theta ) \big |^t \, d\theta - \text {Re}(I_j) = O(t^{-2/5}) \int _{\mathcal {C}} \big |\Phi (\theta ) \big |^t \, d\theta . \end{aligned}$$

The idea is to choose $\varphi$ as a function of *z* and *t* in such a way that the linear term in the Taylor expansion of $\Phi (\theta ) e^{- i z \cdot \theta /t}$ around $\theta ^{j}$ vanishes, i.e.,

$$\begin{aligned} \Phi (\theta ^j) \nabla \left( e^{-i z \cdot \theta ^j/t} \right) + e^{- i z \cdot \theta ^j/t} \nabla \Phi (\theta ^j) = 0. \end{aligned}$$

If we denote the *k*th component of *z* by $z_k$, this is equivalent to

$$\begin{aligned} \frac{z_k}{t} = \frac{\sinh (\varphi _k)}{d \Phi (0)} = \frac{\sinh (\varphi _k)}{\sum _{l=1}^d \cosh (\varphi _l)}, \quad 1 \le k \le d, \end{aligned}$$

by virtue of (A2). Let $F:\mathbb {R}^d \rightarrow \mathbb {R}^d$ be given by

$$\begin{aligned} F(x_1, \ldots , x_d) {:}=\sum _{k=1}^d \frac{\sinh (x_k)}{\sum _{l=1}^d \cosh (x_l)}e_k. \end{aligned}$$

For $r > 0$ and $x \in \mathbb {R}^d$, let $B_r(x)$ denote the open Euclidean ball of radius *r* centered at *x*. Since $F(0) = 0$ and

$$\begin{aligned} \det DF(0) = \frac{1}{d^d} \ne 0, \end{aligned}$$

the Inverse Function Theorem yields existence of $\rho _1 > 0$ and an open neighborhood *U* of 0 such that $F: U \rightarrow B_{\rho _1}(0)$ is a diffeomorphism. Therefore, for every $t \in \mathbb {N}$ and $z \in \mathbb {Z}^d$ with $\Vert z\Vert < \rho _1 t$, there exists $\varphi \in U$ such that $F(\varphi ) = z/t$. Since $F^{-1}$ is differentiable and $F^{-1} (0) = 0$, there exists $\rho _2 >0$ such that

$$\begin{aligned} \Vert \varphi \Vert = \Vert F^{-1} (z/t) \Vert \le \rho _2 \frac{\Vert z\Vert }{t}. \end{aligned}$$

Without loss of generality, we may assume that $\rho _1 \rho _2 \le 1$ so that $\Vert \varphi \Vert \le 1$.

Fix $t \in \mathbb {N}$, $z \in \mathbb {Z}^d$ such that $\Vert z\Vert \le \rho _1 t$ and $q^z_t > 0$, and the corresponding $\varphi \in \mathbb {R}^d$ such that $F(\varphi ) = z/t$. We identify $\varphi$ with the linear functional mapping $e_k$ to $\varphi _k$ for $1 \le k \le d$. For this choice of $\varphi$, the linear term in the Taylor expansion of $\Phi (\theta ) e^{-i z \cdot \theta /t}$ vanishes, so we have for $j \in \{0,1\}$ and $\theta \in {\mathcal {B}}_j$ ($\Vert \theta - \theta ^j \Vert \le t^{-\frac{2}{5}}$)

A6 $$\begin{aligned} \Phi (\theta ) e^{-i z \cdot \theta /t}&= \Phi (\theta ^j) e^{-i z \cdot \theta ^j/t} + (\theta - \theta ^j) \cdot A_j (\theta - \theta ^j) + O \big ( \Vert \theta - \theta ^j \Vert ^3 \big ) \nonumber \\&= \Phi (\theta ^j) e^{- i z \cdot \theta ^j/t} \left( 1 + \frac{(\theta - \theta ^j) \cdot A_j (\theta - \theta ^j)}{\Phi (\theta ^j) e^{- i z \cdot \theta ^j/t}} + O (t^{-6/5}) \right) , \end{aligned}$$

where $A_j$ is the quadratic form in the Taylor expansion of $\Phi (\theta ) e^{-i z \cdot \theta /t}$. The error term $O(t^{-6/5})$ is complex-valued, whereas the entries of $A_j \big / \Phi (\theta ^j) e^{-i z \cdot \theta ^j/t}$ are real numbers. Let $x_j(\theta )$ and $y_j(\theta )$ denote respectively the real and imaginary part of

$$\begin{aligned} 1 + \frac{(\theta - \theta ^j) \cdot A_j (\theta - \theta ^j)}{\Phi (\theta ^j) e^{-i z \cdot \theta ^j/t}} + O(t^{-6/5}). \end{aligned}$$

Then the left-hand side of (A5) can be written as follows:

A7 $$\begin{aligned} \frac{\Phi (0)^t}{(2\pi )^d} \int _{{\mathcal {B}}_j} \bigg ( \big |x_j (\theta ) + i y_j (\theta ) \big |^t - \text {Re}\Big ( \big ( x_j (\theta ) + i y_j (\theta ) \big )^t \Big ) \bigg ) \, d\theta . \end{aligned}$$

Here, in the case $j=1$, we used the assumption that $q^z_t > 0$ and hence *t* and $\Vert z\Vert _1$ have the same parity: as $t \equiv \Vert z \Vert _1$, one has $\Phi (\theta ^1)^t e^{-i z \cdot \theta ^1} = \Phi (0)^t (-1)^t e^{- i \pi \Vert z \Vert _1} = \Phi (0)^t$. If we represent $x_j(\theta ) + i y_j(\theta )$ in polar form, then the modulus is $\big |\Phi (\theta ) / \Phi (0) \big |$ and the argument is of order $O (t^{-6/5})$. As a result, the integrand in (A7) can be written as

$$\begin{aligned} \frac{ \big |\Phi (\theta ) \big |^t}{\Phi (0)^t} \Big (1 - \cos \big (O(t^{-1/5}) \big ) \Big ) = \frac{ \big |\Phi (\theta ) \big |^t}{\Phi (0)^t} O (t^{-2/5}), \end{aligned}$$

which yields (A5).

We continue estimating the expression on the right-hand side of (A4) by showing that for $F(\varphi ) = z/t$, one also has

A8 $$\begin{aligned} \frac{2}{(2 \pi )^d} \int _{\mathcal {C}\setminus ({\mathcal {B}}_0 \cup {\mathcal {B}}_1)} \big |\Phi (\theta ) \big |^t \, d\theta \lesssim t^{-2/5} \int _{\mathcal {C}} \big |\Phi (\theta ) \big |^t \, d\theta . \end{aligned}$$

By Claim A.2, there exist $\varepsilon , \delta > 0$ such that the left-hand side of (A8) is dominated by

$$\begin{aligned} \frac{2 \Phi (0)^t}{(2\pi )^d} \left( \int _{\mathcal {C}\setminus ({\mathcal {B}}_0 \cup {\mathcal {D}}_1^\varepsilon )} \hspace{-3mm} e^{- \delta t \Vert \theta \Vert ^2} d\theta + \int _{\mathcal {C}\setminus ({\mathcal {B}}_1 \cup {\mathcal {D}}_0^\varepsilon )} \hspace{-3mm} e^{- \delta t \Vert \theta - \theta ^1 \Vert ^2} d\theta \right) \lesssim \Phi (0)^t e^{- \delta t^{1/5}} \hspace{-1mm}. \end{aligned}$$

Here we used that

$$\begin{aligned} \mathcal {C}\setminus ({\mathcal {B}}_0 \cup {\mathcal {B}}_1) \subseteq \mathcal {C}\setminus \big [ ({\mathcal {B}}_0 \cap {\mathcal {D}}^{\varepsilon }_0) \cup ({\mathcal {B}}_1 \cap {\mathcal {D}}^{\varepsilon }_1) \big ] \subseteq \big [ {\mathcal {C}} \setminus ({\mathcal {B}}_0 \cup {\mathcal {D}}^{\varepsilon }_1) \big ] \cup \big [ {\mathcal {C}} \setminus ({\mathcal {B}}_1 \cup {\mathcal {D}}^{\varepsilon }_0) \big ]. \end{aligned}$$

As $e^{-\delta t^{1/5}} \lesssim t^{-2/5} t^{-d/2}$, the estimate in (A8) follows once we show that

A9 $$\begin{aligned} J_t {:}=\int _{\mathcal {C}} \left( \frac{|\Phi (\theta ) |}{\Phi (0)} \right) ^t \, d\theta \gtrsim t^{-d/2}. \end{aligned}$$

We have

$$\begin{aligned} J_t&\ge \frac{1}{\Phi (0)^t} \int _{{\mathcal {B}}_0} \big |\Phi (\theta ) e^{-i z \cdot \theta /t} \big |^t \, d \theta = \int _{{\mathcal {B}}_0} \big |x_0 (\theta ) + i y_0 (\theta ) \big |^t \, d\theta \\&\ge \cos \big ( O (t^{-1/5}) \big ) \int _{{\mathcal {B}}_0} \big |x_0 (\theta ) \big |^t \, d \theta = \big ( 1 + O (t^{-2/5}) \big ) \int _{{\mathcal {B}}_0} \big |x_0 (\theta ) \big |^t \, d \theta , \end{aligned}$$

where we used (A6). For $\theta \in {\mathcal {B}}_0$, one has $x_0 (\theta ) = \exp \big ((\theta \cdot A_0 \theta ) / \Phi (0) \big ) \big (1 + O (t^{-6/5}) \big )$, so we can continue the above chain of inequalities as follows:

$$\begin{aligned} J_t&\ge \big ( 1 + O (t^{-2/5}) \big ) \big ( 1 + O (t^{-6/5}) \big )^t \int _{{\mathcal {B}}_0} \exp \left( t \frac{\theta \cdot A_0 \theta }{\Phi (0)} \right) \, d \theta \\&\gtrsim \int _{{\mathcal {B}}_0} \exp \left( t \frac{\theta \cdot A_0 \theta }{\Phi (0)} \right) \, d \theta \gtrsim \int _{{\mathcal {B}}_0} e^{-c t \Vert \theta \Vert ^2 } \, d \theta \gtrsim t^{-d/2}, \end{aligned}$$

where $c > 0$ is some constant. Combining (A4), (A5), and (A8) yields

A10 $$\begin{aligned} q_t^z e^{\varphi (z)} \ge \left( 1 + O(t^{-2/5}) \right) \frac{1}{(2 \pi )^d} \int _{\mathcal {C}} \big |\Phi (\theta ) \big |^t \, d\theta \end{aligned}$$

and hence

$$\begin{aligned} \frac{1}{(2 \pi )^d} \int _{\mathcal {C}} \big |\Phi (\theta ) \big |^t \, d\theta \le \left( 1 + O(t^{-2/5}) \right) q^z_t e^{\varphi (z)}. \end{aligned}$$

To show that

$$\begin{aligned} q^z_t e^{\varphi (z)} \gtrsim t^{-\frac{d}{2}} \sum _{y \in \mathbb {Z}^d} q^y_t e^{\varphi (y)}, \end{aligned}$$

one simply combines (A10) with (A9) and (17).

### A.4 Proof of Lemma 9

Let $\rho {:}=\rho _1$ and $\rho _2$ be as in Lemma 8, and let $t, t' \in \mathbb {N}$, $z, z' \in \mathbb {Z}^d$ such that $\Vert z\Vert \le \rho t$ and $q^z_t > 0$. Let $\varphi$ be the linear functional from Lemma 8 that corresponds to *t* and *z*, and for which $\Vert \varphi \Vert \le \rho _2 \Vert z\Vert /t$ and

A11 $$\begin{aligned} \frac{1}{(2 \pi )^d} \int _{\mathcal {C}} |\Phi (\theta ) |^t \ d \theta \le \left( 1 + O(t^{-\frac{2}{5}}) \right) q_t^z e^{\varphi (z)}. \end{aligned}$$

We consider two cases: $t' > t$ and $t' \le t$.

Case “$t' > t$”. By (A3) and (16), one has

$$\begin{aligned} q_{t'}^{z'} e^{\varphi (z')} \le \frac{1}{(2 \pi )^{d}} \int _{\mathcal {C}} |\Phi (\theta ) |^{t'} \ d \theta \le \Phi (0)^{t'-t} \frac{1}{(2 \pi )^d} \int _{\mathcal {C}} |\Phi (\theta ) |^t \ d \theta . \end{aligned}$$

Furthermore,

$$\begin{aligned} \Phi (0)^{t'-t} \le e^{\Vert \varphi \Vert (t'-t)} \le e^{\rho _2 \Vert z\Vert (t'-t)/t}. \end{aligned}$$

The estimate in (A11) then implies

$$\begin{aligned} \frac{q_{t'}^{z'}}{q_t^z} \le&\left( 1 + O\left( t^{-\frac{2}{5}}\right) \right) e^{\varphi (z-z')} e^{\rho _2 \Vert z\Vert (t'-t)/t} \\ \le&\left( 1+O\left( t^{-\frac{2}{5}}\right) \right) \exp \left( \rho _2 \frac{\Vert z\Vert }{t} \left( \Vert z-z'\Vert + |t' - t |\right) \right) . \end{aligned}$$

Case “$t' \le t$”. If $t' \le t$, the function $x \mapsto x^{t/ t'}$ is convex, and Jensen’s Inequality implies

A12 $$\begin{aligned} q_{t'}^{z'} e^{\varphi (z')} \le \biggl ( \frac{1}{(2 \pi )^d} \int _{\mathcal {C}} |\Phi (\theta ) |^t \ d \theta \biggr )^{t'/t} = \Phi (0)^{t'} J_t^{t'/t} \le \Phi (0)^t J_t^{t'/t}, \end{aligned}$$

where $J_t$ was defined in (A9). Since $J_t \gtrsim t^{-d/2}$,

A13 $$\begin{aligned} J_t^{t'/t} \le \left( c_2 t^{\frac{d}{2}} \right) ^{(t-t')/t} J_t \le \exp \left( c_3 \ln (t) \frac{t-t'}{t} \right) J_t \end{aligned}$$

for some constants $c_2, c_3>0$. Combining (A12) and (A13), we obtain

$$\begin{aligned} q_{t'}^{z'} \le e^{-\varphi (z')} \frac{1}{(2 \pi )^d} \int _{\mathcal {C}} |\Phi (\theta ) |^t \ d \theta \ \exp \left( c_3 \ln (t) \frac{t-t'}{t} \right) . \end{aligned}$$

Together with (A11), this yields

$$\begin{aligned} \frac{q_{t'}^{z'}}{q_t^z} \le&\left( 1 + O(t^{-\frac{2}{5}}) \right) \exp \left( \varphi (z - z')+ c_3 \ln (t) \frac{t - t'}{t} \right) \\ \le&\left( 1 + O(t^{-\frac{2}{5}}) \right) \exp \left( c \left( \frac{\Vert z\Vert }{t} \Vert z - z' \Vert + \ln (t) \frac{t -t'}{t} \right) \right) \end{aligned}$$

for some constant $c > 0$.

## Appendix B A Calculus Estimate

### Lemma 14

There exists $c > 0$ such that for every $t \in \mathbb {N}$, $l \in \mathbb {N}_0$, and $M > 0$,

B14 $$\begin{aligned} \sum _{\begin{array}{c} t_1 + \cdots + t_{l+1} = t, \\ t_1, \ldots , t_{l+1} \ge M \end{array}} \prod _{j=1}^{l+1} t_j^{-\frac{d}{2}} \le \frac{c^l}{M^{l\left( \frac{d}{2}-1\right) }} t^{-\frac{d}{2}}. \end{aligned}$$

The sum on the left-hand side is taken over all positive integers $t_1, \ldots , t_{l+1}$ that satisfy the two conditions under the summation sign.

### Proof

We choose

$$\begin{aligned} c {:}=2^d \max \left\{ \zeta \left( \tfrac{d}{2}\right) , \left( \tfrac{d}{2}-1\right) ^{-1}\right\} , \end{aligned}$$

where $\zeta$ is the Riemann Zeta Function, and prove the statement by induction. In the base case $l=0$, the left-hand side of (B14) is either zero (if $t < M$), or becomes

$$\begin{aligned} t^{-\frac{d}{2}} = \frac{c^0}{M^0} t^{-\frac{d}{2}}. \end{aligned}$$

In the induction step, suppose that (B14) holds for some $l \in \mathbb {N}_0$. Then,

B15 $$\begin{aligned} \sum _{\begin{array}{c} t_1 + \cdots + t_{l+2} = t, \\ t_1, \ldots , t_{l+2} \ge M \end{array}} \prod _{j=1}^{l+2} t_j^{-\frac{d}{2}} = \sum _{\begin{array}{c} t' + t_{l+2} = t, \\ t', t_{l+2} \ge M \end{array}} \biggl ( \sum _{\begin{array}{c} t_1 + \cdots + t_{l+1} = t', \\ t_1, \ldots , t_{l+1} \ge M \end{array}} \prod _{j=1}^{l+1} t_j^{-\frac{d}{2}} \biggr ) t_{l+2}^{-\frac{d}{2}}. \end{aligned}$$

For every $$t'$$,

$$\begin{aligned} \sum _{\begin{array}{c} t_1 + \cdots + t_{l+1} = t', \\ t_1, \ldots , t_{l+1} \ge M \end{array}} \prod _{j=1}^{l+1} t_j^{-\frac{d}{2}} \le \frac{c^l}{M^{l \left( \frac{d}{2}-1\right) }} (t')^{-\frac{d}{2}} \end{aligned}$$

by induction hypothesis. Hence, the right-hand side of (B15) is bounded from above by

B16 $$\begin{aligned} \frac{c^l}{M^{l\left( \frac{d}{2}-1\right) }} \sum _{\begin{array}{c} t' + t_{l+2} = t, \\ t', t_{l+2} \ge M \end{array}} (t')^{-\frac{d}{2}} t_{l+2}^{-\frac{d}{2}}. \end{aligned}$$

We have

$$\begin{aligned} \sum _{\begin{array}{c} t' + t_{l+2} = t, \\ t', t_{l+2} \ge M \end{array}} (t')^{-\frac{d}{2}} t_{l+2}^{-\frac{d}{2}} \le 2 \sum _{\begin{array}{c} t' + t_{l+2} = t, \\ t' \ge t_{l+2} \ge M \end{array}} (t')^{-\frac{d}{2}} t_{l+2}^{-\frac{d}{2}}. \end{aligned}$$

If $t' + t_{l+2} = t$ and $t' \ge t_{l+2}$, it follows that $t' \ge \tfrac{t}{2}$, so the expression on the right-hand side is bounded from above by

B17 $$\begin{aligned} 2^{\frac{d}{2}+1} t^{-\frac{d}{2}} \sum _{t_{l+2} \ge M} t_{l+2}^{-\frac{d}{2}}. \end{aligned}$$

If $M \ge 2$, we have

$$\begin{aligned} \sum _{t_{l+2} \ge M} t_{l+2}^{-\frac{d}{2}} \le \int _{\frac{M}{2}}^{\infty } x^{-\frac{d}{2}} \ dx = \frac{2^{\frac{d}{2}-1}}{\frac{d}{2}-1} M^{1 - \frac{d}{2}}. \end{aligned}$$

If $$M < 2$$,

$$\begin{aligned} \sum _{t_{l+2} \ge M} t_{l+2}^{-\frac{d}{2}} \le \zeta (\tfrac{d}{2}) < \zeta (\tfrac{d}{2}) 2^{\frac{d}{2}-1} M^{1-\frac{d}{2}}. \end{aligned}$$

The expression in (B17) is therefore less than $c M^{1-\frac{d}{2}} t^{- \frac{d}{2}}$. Combining this estimate with (B16) yields

$$\begin{aligned} \sum _{\begin{array}{c} t_1 + \cdots + t_{l+2} = t, \\ t_1, \ldots , t_{l+2} \ge M \end{array}} \prod _{j=1}^{l+2} t_j^{-\frac{d}{2}} \le \frac{c^{l+1}}{M^{(l+1) (\frac{d}{2}-1)}} t^{-\frac{d}{2}}. \end{aligned}$$

$$\square $$
